# Checkpoints couple transcription network oscillator dynamics to cell-cycle progression

**DOI:** 10.1186/s13059-014-0446-7

**Published:** 2014-09-05

**Authors:** Sara L Bristow, Adam R Leman, Laura A Simmons Kovacs, Anastasia Deckard, John Harer, Steven B Haase

**Affiliations:** Department of Biology, Duke University, Durham, NC USA; Duke Center for Systems Biology, Duke University, Durham, NC USA; Program in Computational Biology and Bioinformatics, Duke University, Durham, NC USA; Department of Mathematics, Duke University, Durham, NC USA

## Abstract

**Background:**

The coupling of cyclin dependent kinases (CDKs) to an intrinsically oscillating network of transcription factors has been proposed to control progression through the cell cycle in budding yeast, *Saccharomyces cerevisiae*. The transcription network regulates the temporal expression of many genes, including cyclins, and drives cell-cycle progression, in part, by generating successive waves of distinct CDK activities that trigger the ordered program of cell-cycle events. Network oscillations continue autonomously in mutant cells arrested by depletion of CDK activities, suggesting the oscillator can be uncoupled from cell-cycle progression. It is not clear what mechanisms, if any, ensure that the network oscillator is restrained when progression in normal cells is delayed or arrested. A recent proposal suggests CDK acts as a master regulator of cell-cycle processes that have the potential for autonomous oscillatory behavior.

**Results:**

Here we find that mitotic CDK is not sufficient for fully inhibiting transcript oscillations in arrested cells. We do find that activation of the DNA replication and spindle assembly checkpoints can fully arrest the network oscillator via overlapping but distinct mechanisms. Further, we demonstrate that the DNA replication checkpoint effector protein, Rad53, acts to arrest a portion of transcript oscillations in addition to its role in halting cell-cycle progression.

**Conclusions:**

Our findings indicate that checkpoint mechanisms, likely via phosphorylation of network transcription factors, maintain coupling of the network oscillator to progression during cell-cycle arrest.

**Electronic supplementary material:**

The online version of this article (doi:10.1186/s13059-014-0446-7) contains supplementary material, which is available to authorized users.

## Background

Successful cell divisions depend on proper temporal ordering of cell-cycle events. The central oscillator driving periodic events in early embryos is based on the activity of the mitotic cyclin/ cyclin dependent kinase (CDK) complex [1,2]. CDKs are also the central components of the oscillator in many contemporary models of yeast and metazoan cell-cycle control. However, more recent models suggest a network of interconnected transcription factors may serve as an underlying cell-cycle oscillator in budding yeast, *Saccharomyces cerevisiae* [3-7]. In the network oscillator model, a transcription factor network controls the temporal program of cell-cycle-regulated transcription, including the cyclin genes [5]. Thus, CDK oscillations are driven by the transcription network [5,6], and successive waves of expression of different cyclin/CDK complexes trigger the ordered events of the cell cycle [8,9]. CDKs act as effectors of the transcription network oscillator to trigger cell-cycle events, and feed back on the transcription network to control aspects of oscillation dynamics [6]. In mutant cells lacking CDK activities, the cell cycle arrests; however, transcriptional oscillations continue, indicating that network oscillations and cell-cycle progression can be uncoupled [5,6].

While the ordering of cell-cycle events is important, the time it takes to complete any particular process can vary [10,11], especially when environmental or physiological conditions perturb processes such as DNA replication or spindle assembly [12]. Is there a mechanism that ensures the transcription network oscillator is restrained when cell-cycle progression has been slowed or arrested, or does the network oscillator continue to free-run and get re-entrained at a later time?

**Figure 1:**
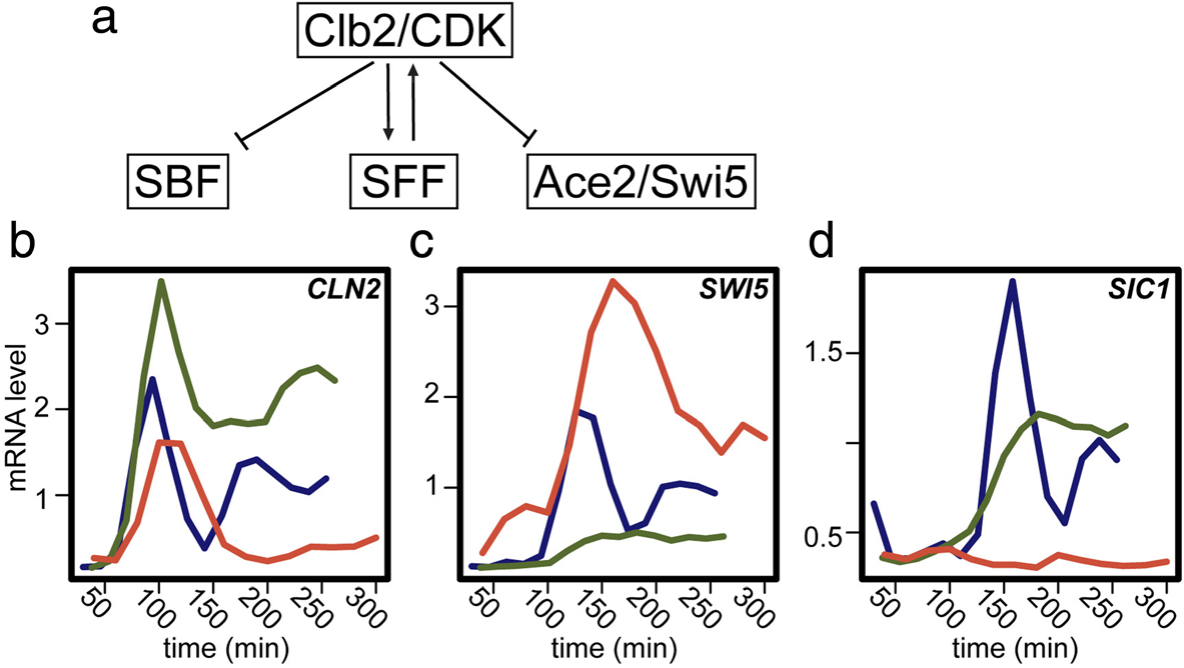
**Persistent Clb2/Cdk1 activity regulates transcript dynamics of network oscillator targets.** A subset of the network oscillator transcription factors are activated and inhibited by Clb2/Cdk1 [14] **(a)**. Absolute mRNA levels (arbitrary expression units) for periodic genes in *P*_*GALL*_ *-CDC20* (CDK ‘on’; red), *∆clb1,2,3,4,5,6* (CDK ‘off’; green), and wild-type (blue) cells **(b, c, d)**. Transcripts are shown for *CLN2* (SBF target) **(b)**, *SWI5* (SFF target) **(c)**, and *SIC1* (Ace2/Swi5) target **(d)**.

It has been proposed that CDK acts as a master oscillator to entrain subordinate autonomous oscillators capable of driving subsets of periodic cell-cycle phenomena [13]. Mitotic CDKs are known to both inhibit and activate specific transcription factors within the network oscillator [14] (Figure 1a), and we have shown that CDKs play a role in controlling oscillation amplitude and period of the network oscillator [6]. In budding yeast, physiological perturbations that inhibit cell-cycle progression do so through checkpoints whose primary effect is thought to be maintenance of high mitotic CDK activity. Therefore, we sought to test the hypothesis that mitotic CDKs function not only as effectors of the network oscillator, but also act to stall the transcription network oscillator when cell-cycle progression is delayed.

## Results

### Persistent Clb2/Cdk1 affects the function of specific network transcription factors

To ask whether persistent levels of mitotic CDK (Clb2/Cdk1) could freeze the network oscillator, we used a strain in which the anaphase promoting complex (APC) activator, Cdc20, is conditionally expressed from a modified *GAL1* promoter (*P*_*GALL*_*-CDC20*) in a *cdc20∆* background [15]. When cells are shifted from galactose to glucose medium, Cdc20 is depleted, arresting cells at the metaphase-to-anaphase transition with persistent levels of Clb2 protein (Additional file 1: Figure S1) and Clb2/Cdk1 activity [16,17]. A G1-synchronized population of *P*_*GALL*_*-CDC20 cdc20∆* cells was collected by centrifugal elutriation, and suspended in dextrose-containing growth medium at time 0. Aliquots of cells were collected at 20-min intervals for 300 or 360 min (two experimental replicates). Genome-wide transcript levels were assayed at each time point by microarray. Cell-cycle progression and subsequent arrest was monitored by observing bud and spindle formation (Additional file 1: Figure S1). Results from two independent replicates were highly reproducible, with an *r*^*2*^ value of 0.98 (Additional file 1: Figure S1).

Clb2/Cdk1 is known to regulate the activity of network transcription factors and complexes including SBF (SCB binding factor), SFF (Swi5 factor), Ace2, and Swi5 [14] (Figure 1a). In the absence of Nrm1, a role for Clb2/Cdk1 in downregulating MBF (MCB binding factor) was also revealed [18]. We compared the dynamic transcript behaviors of SBF-, SFF-, Swi5-, and Ace2-regulated genes from arrested cells depleted of Cdc20 (*P*_*GALL*_*-CDC20;cdc20Δ*; CDK ‘on’), arrested cells depleted of mitotic and S-phase cyclins (*clb1,2,3,4,5,6*;CDK ‘off’) cells, and normally cycling wild-type (WT) cells. Consistent with the known regulatory interactions between these network transcription factors and Clb2/Cdk1, genes regulated by these transcription factors behave differently across the conditions (Figure 1b-d). Transcripts from SBF-regulated G1/S genes are repressed by Clb2/Cdk1 after the first cycle of expression in Cdc20-depleted cells and are expressed at elevated levels, continuing to oscillate, in cyclin mutant cells (Figure 1b). Many transcripts from G2/M genes regulated by SFF are expressed at persistent or elevated levels in Cdc20-depleted cells, likely due to positive feedback with Clb2 [14] (Figure 1c). M/G1 genes regulated by the transcription factors Ace2 and Swi5 are not expressed in Cdc20-depleted cells, likely because Clb2-dependent phosphorylation of Swi5 and Ace2 sequesters the transcription factors in the cytoplasm [14] (Figure 1d).

### Network oscillations continue in the presence of persistent Clb2/Cdk1 activity

**Figure 2:**
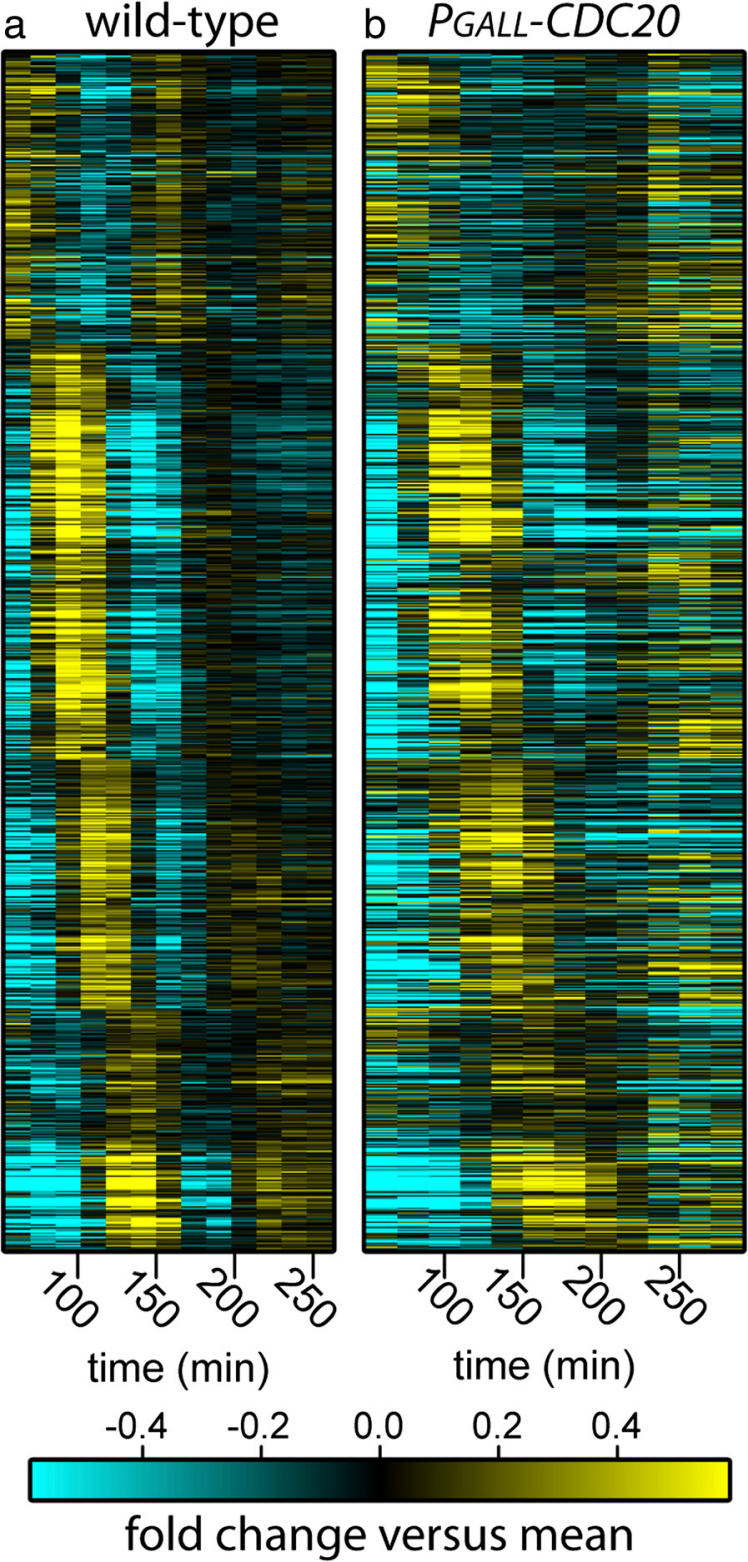
**Dynamics of periodic transcripts in arrested cells with CDK-‘on’ and cycling WT cells.** Heat maps depict mRNA levels of a set of 856 periodic genes in cycling WT **(a)** and arrested CDK ‘on’ (*P*_*GALL*_ *-CDC20;cdc20*) cells **(b)**. Transcript levels are depicted as log_2_-fold change relative to the mean expression of each gene. Each row represents the same gene in both conditions (Additional file 3). Each column represents a time point.

**Figure 3:**
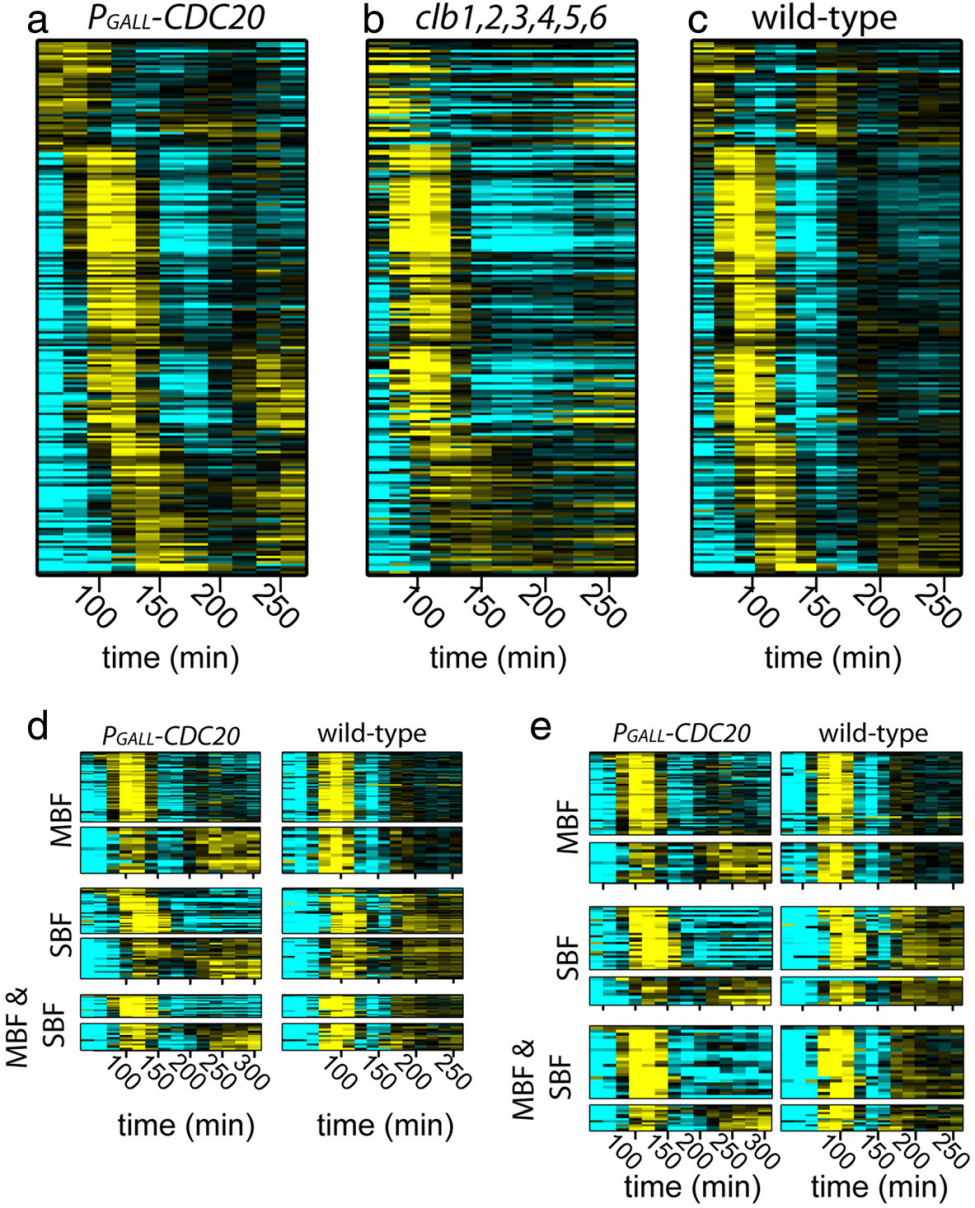
**Dynamics of transcripts in Cdc20-depleted cells, cyclin-depleted cells, and cycling WT cells.** Heat maps depicting mRNA levels of 208 genes that remain periodic in cells arrested with CDK ‘on’ (*P*_*GALL*_ *-CDC20;cdc20)* **(a)**. Heat maps show behaviors of the same genes in arrested cells with CDK ‘off’ (*∆clb1,2,3,4,5,6*) **(b)**, and in normally-cycling WT **(c)** cells. Each row represents the same gene in all three conditions (Additional file 3). Heat maps depicting mRNA levels of SBF- and MBF-regulated genes from annotated lists generated from two separate studies: Ferrezuelo et al. [24] **(d)** and Eser et al. [23] **(e)** in CDK ‘on’ (left) and WT (right) cells. Transcript levels are depicted as log_2_-fold change relative to the mean expression of each gene and are depicted as in Figure 2. Each column represents a time point.

We first defined a set of high confidence periodic genes from WT cells using the intersection of the outputs of multiple periodicity-finding algorithms (see Additional files 1 and 2, Supplementary methods for methods and rationale) [19]. By examining this set of 856 high-confidence periodic genes (Additional file 3), it is clear that oscillations of many transcripts are halted in cells arrested by depletion of Cdc20 (CDK ‘on’) and yet many transcripts continue to oscillate (Figure 2) [19]. Surprisingly, we found that 206 out of 856 genes identified as periodic in WT cells continue to oscillate in arrested CDK ‘on’ cells with dynamics similar to those observed in WT cells (Figures 2 and 3a,c; Additional file 3). Furthermore, of the 206 transcripts that oscillate in Cdc20-depleted cells, approximately 40% also oscillate in cyclin mutant cells (Figure 3a and b). Strikingly, the period of oscillations is nearly identical between the Cdc20-depleted (CDK ‘on’) cells and the cyclin mutant (CDK ‘off’) cells [6]. Taken together, these results demonstrate that while persistent mitotic CDK activity does arrest a portion of the periodic transcription program, the transcriptional network can still produce oscillations with a period similar to those observed in cells depleted for mitotic CDK activity.

Although a substantial fraction of transcripts continue to oscillate in cells depleted of Cdc20, approximately 75% of cycling genes cease to be periodic (Figure 2). How then does the network oscillator maintain oscillations while several of its component transcription factors are inhibited by CDK activity? Many periodic genes in normally cycling cells are regulated by SBF. Because Clb2 is known to inhibit SBF-mediated transcription [8], these genes should not oscillate in cells arrested with high CDK activity. Oscillations could continue through a parallel branch of the network containing the related transcription factor complex, MBF (Additional file 1: Figure S2) [20,21], but mitotic cyclin/CDK has also been shown to downregulate MBF-regulated genes in cells lacking the co-repressor, Nrm1 [18].

In order to determine whether oscillations are perpetuated via the MBF or SBF branch of the network in Cdc20-depleted cells, we closely examined the behaviors of genes that have been annotated as MBF- or SBF-regulated. MBF and SBF have distinct but overlapping sets of target genes, and genetic studies indicate that they are redundantly required for viability [22-25]. Annotated gene sets from two separate studies [23,24] were clustered based on transcript behaviors in Cdc20-depleted cells (Figure 3d and e). Regardless of the gene list, some genes (annotated as SBF-regulated, MBF-regulated, or both) exhibited oscillating transcripts, while others did not (Figure 3d and e). Although most genes annotated as MBF-regulated exhibited strong or damped oscillations as expected (Figure 3d and e, top panels), it was surprising to see that a substantial fraction of SBF-regulated transcripts also oscillated (Figure 3d and e, middle panels). Moreover, genes annotated as both SBF- and MBF-regulated also exhibited a substantial fraction of oscillating genes (Figure 3d and e, bottom panels). Thus, it is possible that oscillations could be propagated on either network branch. Alternatively, oscillations could be driven by transcription factors that bind in addition to SBF or MBF. An overrepresented transcription factor analysis reveals that clusters of genes with different behaviors in Cdc20-depleted cells do indeed have the potential for additional regulation and that distinct regulators may contribute to altered behaviors (Additional file 1: Figure S3 and Table S1). Certainly, further functional analyses will be required to fully understand the behaviors of genes regulated by SBF and MBF across different conditions.

In Cdc20-depleted cells, we find that targets of all transcription factors of the network oscillator [5,6] except for SBF, Ace2, and Swi5 maintain oscillatory behavior (Additional file 1: Figure S2). Thus, the oscillatory function of the network may be supported by redundant sets of transcription factors. Consistent with this finding, several individual transcription factors within the network oscillator can be perturbed without abrogating transcriptional oscillations [6].

Our data clearly demonstrate that persistent Clb2/Cdk1 activity is not sufficient to inhibit the oscillatory function of the entire network during cell-cycle arrest. Moreover, we have observed that transcriptional oscillations occur in cells arrested by mutation of mitotic and S-phase cyclins [5,6] (Figures 2 and 3). These findings beg the question: do network oscillations always continue regardless of cell-cycle arrest or progression, or are there mechanisms in addition to mitotic CDK that may be necessary to arrest the network oscillator during physiological cell-cycle arrests?

### Checkpoint pathways stall the transcription network oscillator

**Figure 4:**
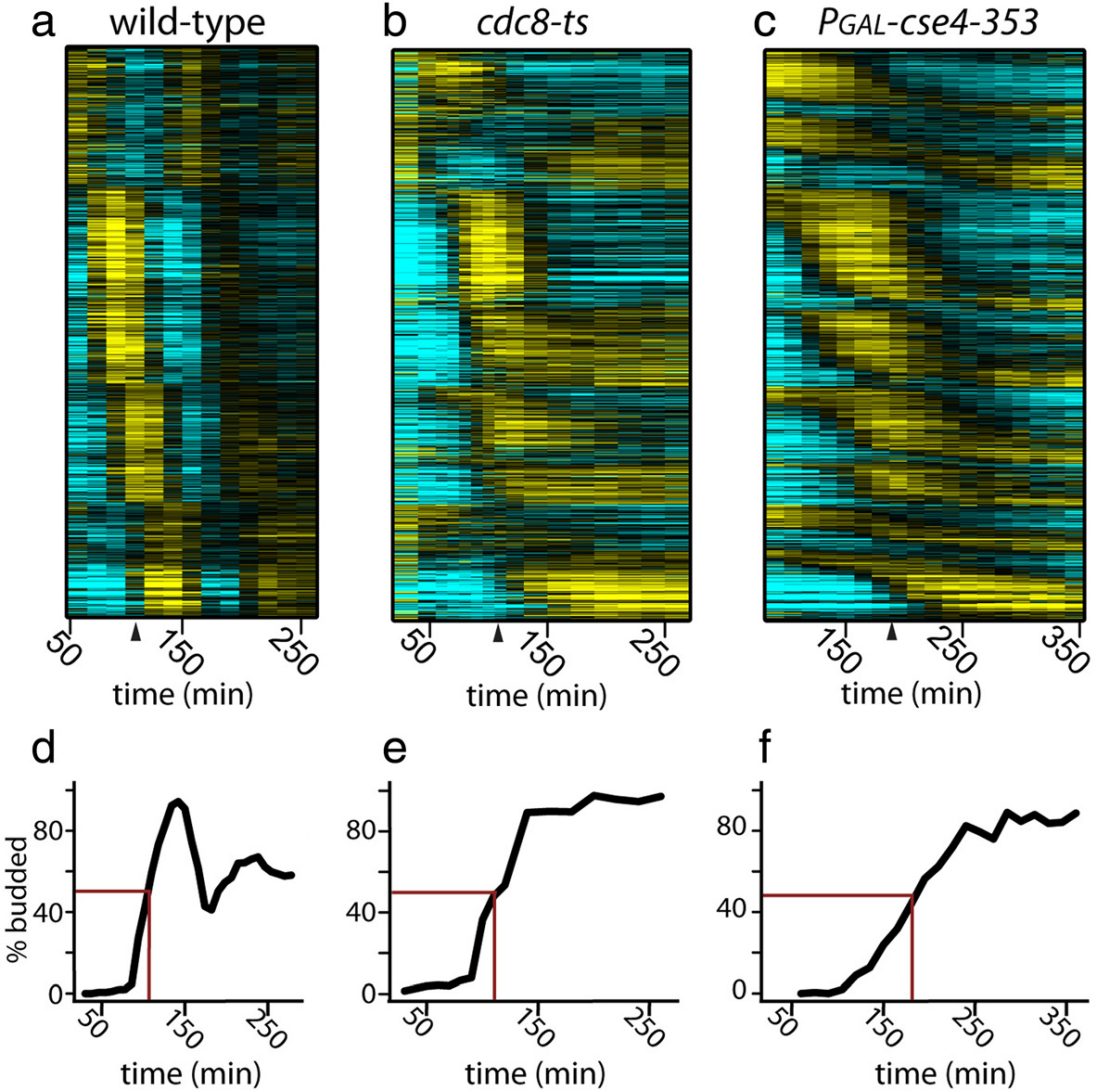
**Dynamics of periodic transcripts are halted during the DNA replication and spindle assembly checkpoints.** Heat maps showing mRNA levels of a set of 856 WT periodic genes in cycling WT cells **(a)**, arrested *cdc8* ^*ts*^ cells (DNA replication checkpoint) **(b)**, and arrested *P*_*GAL*_ *-cse4-353* cells (spindle assembly checkpoint) **(c)**. The same order of genes is shown in all conditions (Additional file 3). Budding indices of WT **(d)**, *cdc8* ^*ts*^ **(e)**, and *GAL1-cse4-353* cells **(f)**. Black line, % budded; red line, 50% one bud **(d-f).**

In normally cycling cells, perturbation of cell-cycle events leads to the activation of checkpoint pathways that subsequently halts cell-cycle progression until events are completed with fidelity [12]. To determine whether cell-cycle checkpoints may be required for a full arrest of transcription factor network oscillations, we measured global transcript dynamics in synchronized populations of cells arrested by two distinct checkpoint mechanisms: the DNA replication checkpoint or spindle assembly checkpoint (Figure 4).

The DNA replication checkpoint was triggered using a temperature sensitive allele of the thymidylate kinase gene, *cdc8*, to deplete nucleotides [26]. The spindle assembly checkpoint was triggered by constitutive over-expression of a mutant allele of the kinetochore protein, *CSE4* (*P*_*GAL1*_*-cse4-353* [27]), which disrupts spindle organization. Checkpoint-mediated cell-cycle arrest was monitored by measuring budding index, and either DNA content or spindle length (Figure 4d, e, and f and Additional file 1: Figure S4). Genome-wide transcript levels were measured by microarray. Results from two independent replicates were highly reproducible for the DNA replication and spindle assembly checkpoints, with an *r*^*2*^ value of 0.99 and 0.93, respectively (Additional file 1: Figure S4).

**Figure 5:**
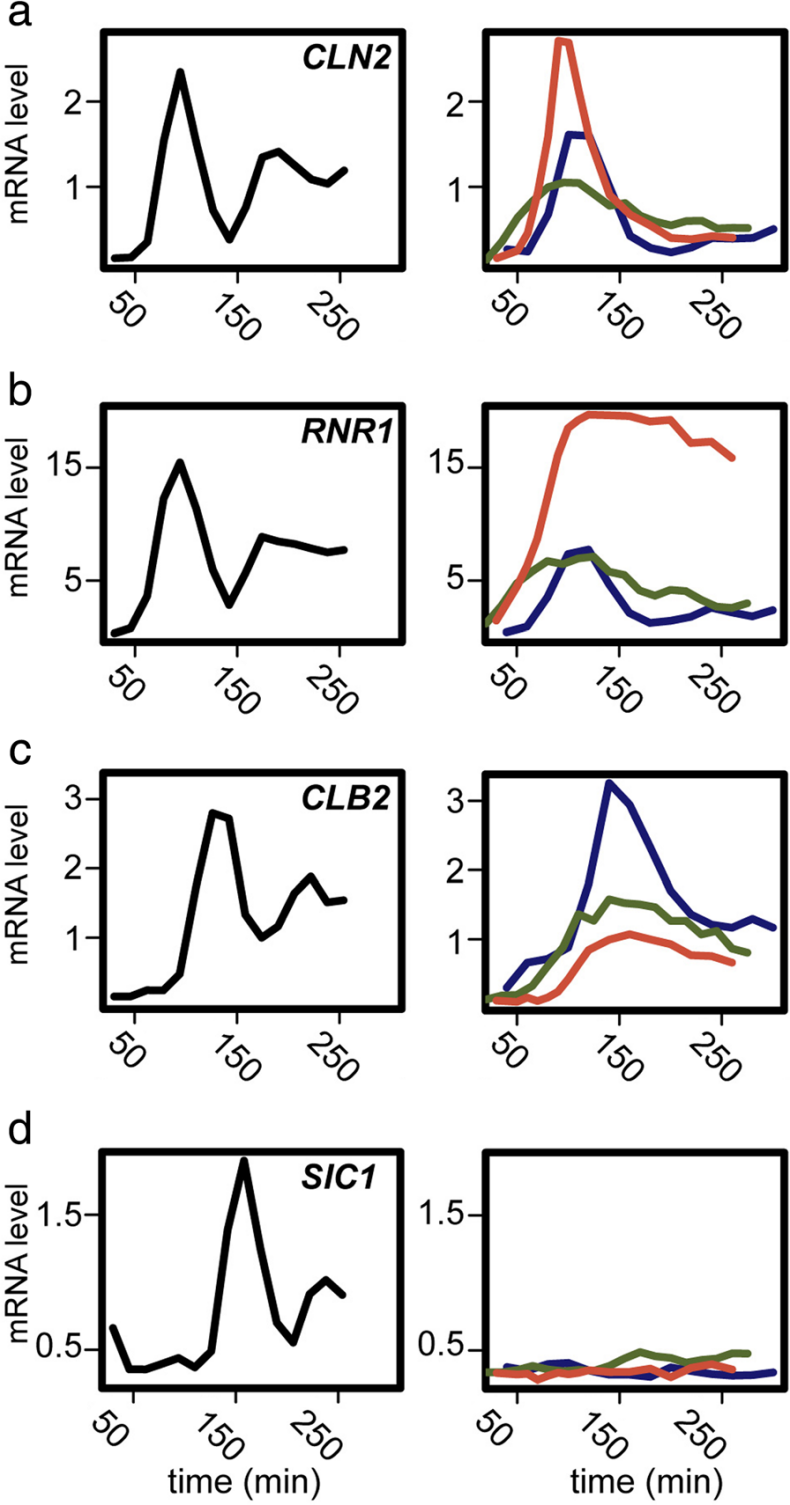
**Oscillating transcripts arrest with distinct behaviors during cell-cycle checkpoints.** Absolute mRNA levels (arbitrary expression units) are shown for periodic genes in normally-cycling WT cells (black; left), Cdc20-depleted cells (CDK ‘on’) (blue; right) DNA replication checkpoint-arrested cells (*cdc8* ^*ts*^) (red; right), and spindle assembly checkpoint-arrested cells (*P*_*GAL*_ *-cse4-353*) (green; right) **(a-d)**. mRNA levels from arrested *cdc8* ^*ts*^ cells (DNA replication checkpoint) and arrested *P*_*GAL*_ *-cse4-353* cells (spindle assembly checkpoint) were temporally aligned on the graph at the point where 50% of the synchronized population had budded. *CLN2* (SBF target) **(a)**, *RNR1* (MBF target) **(b)**, *CLB2* (SFF target) **(c)**, and *SIC1* (Ace2/Swi5 target).

Previous genomic studies utilizing non-synchronized cells identified only a handful of transcripts that appeared to be regulated by the DNA replication or damage checkpoints [28], yet we observe that nearly the entire cell-cycle-regulated transcriptional program appears to halt in response to these checkpoints (Figure 4a-c, Additional file 1: Figure S5 and S6). Our ability to identify these broad changes in transcriptional dynamics likely reflects the use of synchronous populations of cells and high-density sampling across a time series. A recent study using synchronous cells and lower density sampling also indicates that the expression of large clusters of cell-cycle regulated genes is affected by activation of the DNA replication checkpoint [29]. Interestingly, we find that transcriptional behaviors at the arrest point are distinct for the two checkpoints (Figure 4b, c; Figure 5).

### Checkpoints arrest oscillatory dynamics with distinct behaviors

Transcript oscillations came to a halt with a variety of different behaviors, suggesting that multiple transcription factors within the network are regulated differentially by each checkpoint (Figure 5). Clues to the mechanisms by which checkpoints control the transcriptional network are evident when examining specific clusters of co-regulated genes. For example, a set of MBF-regulated genes is persistently expressed during the DNA replication checkpoint (Additional file 1: Figure S7 and S8). By contrast, MBF-regulated genes are expressed for one cycle and subsequently repressed during the spindle assembly checkpoint (Figure 5b). Persistent expression of MBF targets during the DNA replication checkpoint is consistent with the behavior of MBF targets during replication checkpoint activation in fission yeast, *Schizosaccharomyces pombe* [30]. Studies in *S. pombe* indicate checkpoint control of MBF activity is mediated by Rad53-dependent regulation of the MBF activator Cdc10 [30] and the co-repressor Nrm1 [31]. Recent reports indicate Rad53 inactivates Nrm1 as part of the DNA replication checkpoint in *S. cerevisiae* [29,32]. Likewise, downregulation of SBF targets in both DNA replication checkpoint- and spindle assembly checkpoint-arrested cells, as in Cdc20-depleted cells, suggest that stabilization of Clb2 by inhibition of APC^Cdc20^ may be responsible for control of this regulon (Figure 5a, Additional file 1: Figure S7 and S8). The positive feedback between Clb2/Cdk1 and SFF results in persistent target expression, but not at elevated levels compared to normally cycling cells or Cdc20-depleted cells (Figure 5c, Additional file 1: Figure S9). This finding indicates some unknown mechanism may modulate the transcript level of SFF-regulated genes. Swi5- and Ace2-regulated clusters exhibit similar transcript dynamics in cells arrested by the two checkpoints, as well as Cdc20-depleted cells (Figure 5d, Additional file 1: Figure S10). Taken together, these observations suggest that persistent Clb2/Cdk1 acts in concert with other checkpoint effectors to fully arrest the network oscillator.

### Rad53, a DNA replication checkpoint effector kinase, arrests transcript oscillations

Rad53, APC^Cdc20^/Clb2-Cdk1, and Dun1 are effector protein kinases known to control transcription in response to activation of the DNA replication checkpoint [28,33]. As much of the DNA replication checkpoint kinase signaling is downstream of Rad53, we asked how ablating the checkpoint-signaling pathway downstream of Rad53 would affect cell-cycle transcriptional oscillations. We measured global transcript dynamics in synchronized populations of cells depleted of Cdc20 (CDK ‘on’; *cdc20;P*_*GALL*_*-CDC20*) and lacking Rad53 activity (*rad53-1*, also known as *rad53-11* [34-37]) during the DNA replication checkpoint (full genotype: *cdc20Δ;P*_*GALL*_*-CDC20;cdc8*^*ts*^*;rad53-1*). Cdc20 was depleted in addition to triggering the checkpoint to prevent cells from progressing through the cell cycle, as Rad53 is necessary for checkpoint-mediated cell-cycle arrest. A G1-synchronized population of *cdc20;P*_*GALL*_*-CDC20;cdc8*^*ts*^*;rad53-1* cells was collected by centrifugal elutriation, and suspended in dextrose-containing growth medium at time 0 min. Aliquots of cells were collected at 18-min intervals for 360 min (two experimental replicates). Genome-wide transcript levels were assayed at each time point by microarray. Cell-cycle progression and subsequent arrest was monitored by observing bud formation (Additional file 1: Figure S11).

Results from two independent replicates were highly reproducible, with an *r*^*2*^ value of 0.99 (Additional file 1: Figure S11). We compared these mRNA expression patterns to a synchronous population of cells arrested by the DNA replication checkpoint with Cdc20 depleted (*cdc8*^*ts*^;*cdc20;P*_*GALL*_*-CDC20*). A G1-synchronized population of *cdc20;P*_*GALL*_*-CDC20;cdc8*^*ts*^ cells was collected and analyzed as described above for *cdc20;P*_*GALL*_*-CDC20;cdc8*^*ts*^*;rad53-1* cells (one experimental replicate). Gene transcript levels were very similar to *cdc8*^*ts*^ replicate one, with an *r*^*2*^ value of 0.92 (Additional file 1: Figure S11).

**Figure 6:**
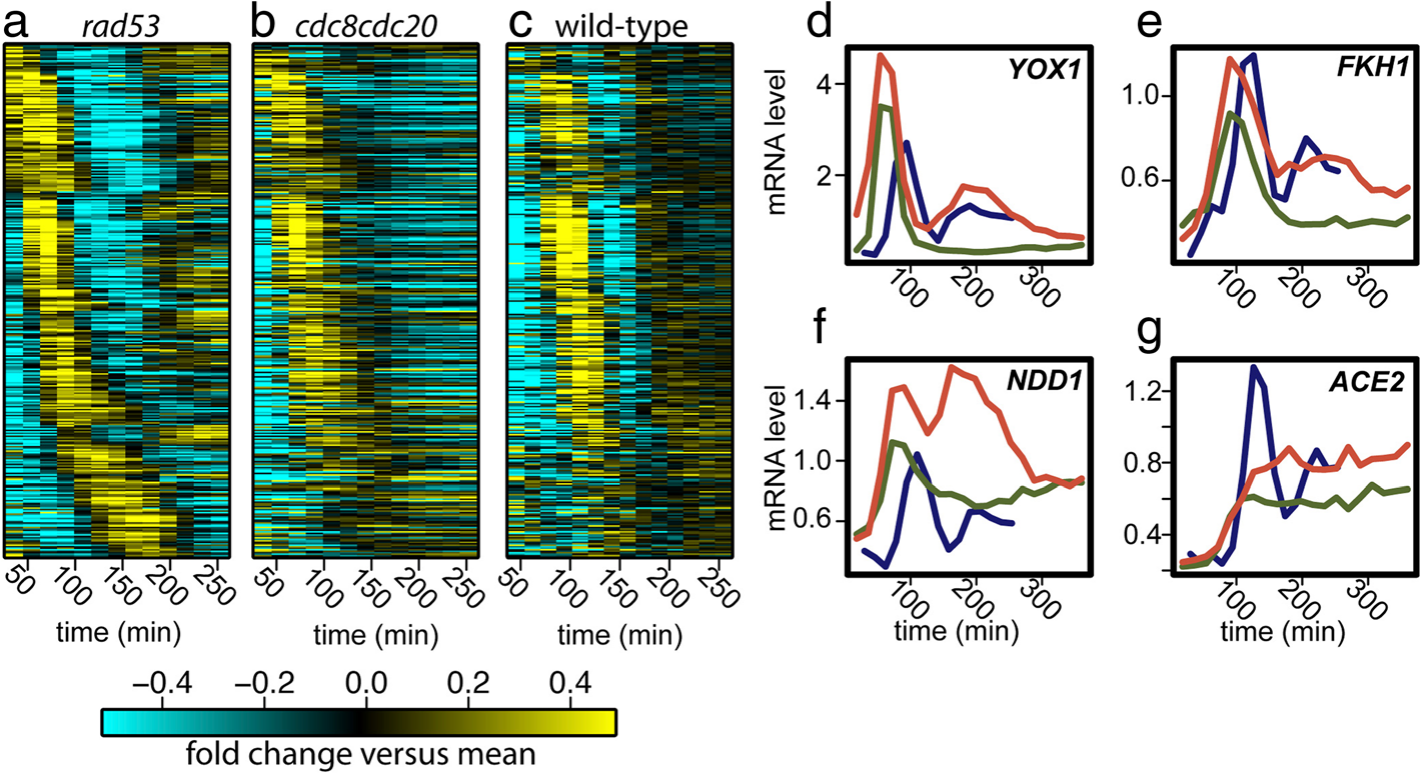
**Periodic transcription is partially restored in the absence of Rad53 activity during the DNA replication checkpoint.** Heat maps showing mRNA levels of a set of 343 WT periodic genes in cell-cycle arrested *cdc8* ^*ts*^ *;P*_*GALL*_ *-CDC20;cdc20;rad53-1* cells (DNA replication checkpoint without Rad53 kinase activity) **(a)**, arrested *cdc8* ^*ts*^ *;cdc20;P*_*GALL*_ *-CDC20* cells (DNA replication checkpoint) **(b)**, and cycling WT cells **(c)**. The same order of genes is shown in all conditions (Additional file 3). Transcript levels are depicted as log_2_-fold change relative to the mean expression and are depicted as in Figure 2. Transcript dynamics are shown for individual genes: *YOX1* **(d)**, *FKH1* **(e)**, *NDD1* **(f)**, and *ACE2* **(g)**. Log_2_-fold mRNA levels for periodic genes in *cdc8* ^*ts*^ *;P*_*GALL*_ *-CDC20;cdc20;rad53-1* (no Rad53 activity; red), *cdc8* ^*ts*^ *;P*_*GALL*_ *-CDC20;cdc20* (DNA replication checkpoint; green), and WT (blue) cells **(d-g)**.

The loss of Rad53 activity during the replication checkpoint restored a substantial portion of periodic transcript oscillations (Figure 6a and b). Although there are virtually no periodic genes in cells arrested in by the DNA replication checkpoint (Figures 4, 5, and 6b), 343 genes out of the set of 856 genes that cycle in WT cells, returned to cycling in cells that lack Rad53 activity (Additional file 3). Moreover, we find that periodic behavior is restored to some but not all cell-cycle transcription factors (Figure 6d-g), yet cell-cycle transcription oscillations are restored on a large scale (Figure 6a). A number of SBF-regulated genes, such as Yox1, become periodic in the absence of Rad53 activity, suggesting that the DNA replication checkpoint kinases are responsible for arresting SBF-mediated transcription in addition to MBF/Nrm1-regulated genes (Figure 6d, Additional file 1: Figure S12).

Taken together, these findings indicate that Rad53 and its downstream effectors are responsible for regulating a large fraction of checkpoint-regulated genes. However, because the transcriptional program does not appear fully WT after the loss of Rad53, it is likely that there are effectors in addition to Rad53 and APC^Cdc20^ acting on the network during the replication checkpoint.

## Discussion

The results presented herein suggest that the transcription network is not simply a CDK-subordinate mechanism that can produce spurious oscillations when CDK activities are limiting. Even when stabilized mitotic CDK inhibits a large portion of the network, it is still capable of driving oscillations with a period nearly identical to the period observed in cells lacking mitotic CDKs (Figures 2 and 3). Consistent with previous observations [6], CDK can modulate various aspects of oscillator function, yet oscillations produced by the transcription network are clearly robust to substantial changes in CDK levels. Moreover, we demonstrate that two distinct checkpoint pathways both act to halt transcript dynamics during a cell-cycle arrest (Figures 4, 5, and 6), suggesting the importance of maintaining coordination between cell-cycle progression and network oscillator dynamics.

In addition to its role in coordinating the DNA-replication checkpoint response, our results indicate that the checkpoint effector kinase, Rad53, collaborates with stabilized mitotic kinases in order to arrest oscillations in the cell-cycle transcription network during the checkpoint (Figure 6). Some of the behaviors observed (Figure 6) could be an indirect effect of Rad53’s regulation of the checkpoint kinase, Dun1 [38,39]. Moreover, the fact that approximately half of the genes in a *rad53-1;cdc8*^*ts*^*;cdc20;P*_*GALL*_*-CDC20* cells resume oscillation (Figure 6a) leaves open the possibility that the checkpoint kinase, Mec1 [37,40] or other checkpoint-dependent mechanisms are regulating the other genes in the cell-cycle transcription network. Further studies will be needed to precisely identify the comprehensive set of checkpoint mechanisms that control the function of the network, and to identify the set of transcription factors that are under regulation. Nonetheless, our results are certainly consistent with reports of Rad53-mediated phosphorylation targeting cell-cycle transcription factors upon activation of the DNA replication checkpoint in *S. cerevisiae* [29,32,35,41,42], and *S. pombe* [30,31,43].

Although the results of our studies (and others) indicate that multiple transcription factors within the cell-cycle-network are targets of the replication checkpoint, SBF and MBF are likely to be important targets based on the fact that they control genes involved in DNA metabolism [24,25]. Mechanisms have been proposed for the regulation of MBF and SBF by Rad53 and mitotic cyclin/CDK, and these mechanisms predict distinct behaviors of SBF- and MBF-regulated genes in cells arrested by the DNA checkpoint [29,32,35]. Just as observed in the Cdc20-depleted cells, the behaviors observed for SBF- and MBF-regulated genes in the *rad53-1;cdc8*^*ts*^*;cdc20;P*_*GALL*_*-CDC20* cells are not fully consistent with expectations (Figure 6a and d and Additional file 1: Figure S12). For example, it is surprising that several SBF-regulated genes resume oscillation in *rad53-1;cdc8*^*ts*^*;cdc20;P*_*GALL*_*-CDC20* cells (Figure 6d and Additional file 1: Figure S12), because persistent Clb2/Cdk1 (due to Cdc20 depletion) should continue to inhibit SBF transcription (Figure 1) even in the absence of Rad53 activity [8]. It is unlikely that these unexpected behaviors are the result of mis-annotation the SBF and MBF gene lists. Analyses of behaviors across multiple checkpoint and Cdc20-depletion conditions illustrates that genes annotated as SBF- or MBF-regulated do not always have consistent and distinct behaviors across all conditions (Figure 3d and e, Additional file 1: Figures S7, S12, and S13). It is likely that there are additional layers of control for SBF- and MBF-regulated transcription that vary across conditions. For example, SBF/MBF switching has been observed during checkpoint responses [32]. It is also possible that additional transcription factors may contribute to the regulation of SBF and MBF targets in different conditions (Additional file 1: Figures S3, S5, and S6 and Tables S1, S2, and S3).

Despite its identification around the same time as the replication checkpoint, the spindle assembly checkpoint targets have not been exhaustively identified [44,45]. Although there are multiple effector kinases in the DNA replication checkpoint pathway that have transcriptional targets, Clb2/Cdk1 in the Cdc20 effector pathway of the spindle assembly checkpoint is the only one known to target transcription factors [8,17,46,47]. Because Cdc20 depletion on its own does not halt oscillations of the entire transcriptional program (Figures 2 and 3), it is clear there are other effector pathways in the spindle assembly checkpoint that are responsible for regulating transcription. Identification of these pathways and targets will further our understanding of how the spindle assembly checkpoint controls the transcriptional network oscillations.

## Conclusion

**Figure 7:**
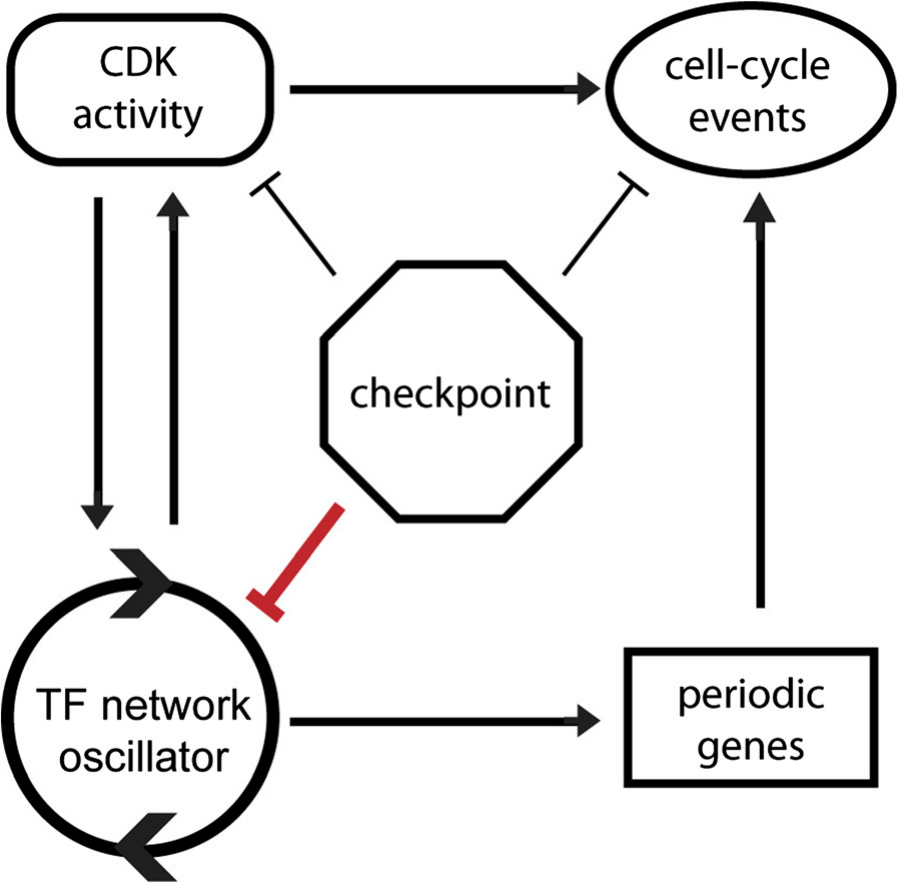
**A new model of cell-cycle regulation.** Cell cycle model depicting relationships between checkpoints, CDKs, transcription network oscillator, periodic genes, and cell-cycle events.

Taken together, our findings suggest that checkpoint pathways evolved to regulate both cell-cycle progression and transcription in order to maintain phase coherence between cell-cycle progression and the transcription network oscillator. Here, we propose a new cell-cycle model in which two functional modules, CDK and checkpoints, are intimately coupled with a transcription network that serves as an underlying cell-cycle oscillator (Figure 7).

## Materials and methods

### Strains and cell synchronization

WT and all mutant strains of *S. cerevisiae* are derivatives of BF264-15Dau and were constructed by standard yeast methods. A description of all yeast strains and plasmids used in this study are outlined in Additional file 1: Table S4. Standard growth conditions were used. Cells were synchronized as previously described [5,6].

### RNA isolation and microarray analysis

For all global mRNA level studies, total RNA was isolated at time intervals by methods described previously [3]. RNA was purified and concentrated using the RNAeasy MinElute Cleanup Kit (QIAGEN). mRNA amplification and fluorescent labeling was done using either the GeneChip One Cycle Labeling (Affymetrix) or the Ambion MessageAmp Premier kit (Ambion Biosystems). Labeled cDNA was hybridized to Yeast 2.0 Expression arrays (Affymetrix) and image collection was carried out by the Duke Microarray Core Facility [48] using standard Affymetrix protocols.

### Data access

Newly generated gene expression microarray data from this manuscript have been submitted to the NCBI Gene Expression Omnibus [49] under accession number GSE49650.

## Additional files

Additional file 1:
**Information and accompanying figures on methods used to identify periodic genes.**
Additional file 2:
**Table listing the deLichtenberg and Lomb-Scargle rankings and** ***P*** **values for identifying periodic genes across all genetic backgrounds.**
Additional file 3:
**Table listing the WT periodic genes based on de Lichtenberg and Lomb-Scargle rankings and whether these genes remain periodic in other genetic backgrounds.**
