# Supplementary material for: Checkpoints couple transcription network oscillator dynamics to cell-cycle progression
Source: Genome Biol. 2014 Sep 5;15(9):446. doi: 10.1186/s13059-014-0446-7 (PMC4180952; doi:10.1186/s13059-014-0446-7)
Supplement: Additional file 1: — Information and accompanying figures on methods used to identify periodic genes. [file 13059_2014_446_MOESM1_ESM.pdf]

Supplementary Information to accompany "Checkpoints Couple Transcription Network Oscillator Dynamics to Cell Cycle Progression"

By Sara L. Bristow, Adam R. Leman, Laura A. Simmons Kovacs, Anastasia Deckard, John Harer, and Steven B. Haase

Additional Methods

Data Normalization

CEL files from the Affymetrix Yeast 2.0 oligonucleotide arrays generated from this study (*cdc20Δ;P_{GALL}-CDC20*, *$cdc8^{ts}$*, *P_{GAL}-cse4-353, $cdc8^{ts}$;cdc20Δ;P_{GALL}-CDC20, and rad53;$cdc8^{ts}$;cdc20Δ;P_{GALL}-CDC20*) and those from previous studies (wild-type and *clb1,2,3,4,5,6*) [1] were normalized and summarized using the dChip [2] method from the `affy` package (v. 1.32.1) in Bioconductor [3] within the R statistical programming environment [4]. The probes specific to *S. pombe* were removed using the s_cerevisiae.msk file from Affymetrix before normalizing the CEL files. The command used to normalize and summarize the CEL files was `expresso(inputdata, normalize.method= ″invariantset″, bgcorrect.method= ″none″, pmcorrect.method= ″pmonly″, summary.method =″liwong″, verbose= TRUE).`

Calculating Wild-Type Cell-Cycle Period and Identifying Periodic Gene Sets

We have previously measured cell-cycle period in wild-type cells using CLOCCS [5]. However, the cell-cycle period output by CLOCCS only takes into account a homogenous population of mother cells, while the actual wild-type population contains a mix of mother cells and daughter cells in the second cell cycle. To account for this heterogeneity, we averaged the mother cell-cycle period (λ) and the daughter cell-cycle period (λ+δ) for each wild-type replicate using the outputs from CLOCCS [1, 5]. The result of this average increased the original cell-cycle period used in analysis from 77.1 minutes in replicate 1 and 85.0 minutes in replicate 2 to 97.8 minutes in replicate 1 and 102.6 minutes in replicate 2.

We applied the deLichtenberg algorithm to the wild-type transcript dynamics data using the new mother/daughter-averaged cell-cycle period [6]. At a p-value cutoff of $p \leq 0.2$, we identified 1,912 wild-type periodic genes (Additional Files 2 and 3). Of the 1,275 periodic genes from

Orlando *et al* [1], 1010 genes were also classified as oscillating at the mother/daughter-averaged cell-cycle period. In addition to a large overlap with Orlando *et al* [1], the averaged cell-cycle period adds 902 periodic genes.

Additionally, periodicity of wild-type gene expression dynamics was scored using the Lomb-Scargle algorithm across a range of periods from 50 minutes to 300 minutes [4, 7, 8]. The output for each wild-type replicate can be found in Additional File 2. We chose a period of 97 minutes with a ten-minute window around that average cell-cycle period, and a p-value cutoff of $p \leq 0.5$. This results in 991 periodic genes in wild-type cells (Additional File 3). Note that the period identified by Lomb-Scargle using expression profiles is very similar to the mother-daughter-averaged period derived from budding index by CLOCCS algorithm above.

Using only genes identified as periodic in both the deLichtenberg and Lomb-Scargle analyses, we generated a high-confidence wild-type periodic gene list. This results in a wild-type periodic gene list containing 856 genes (Figures 2 and 4, Additional Files 2 and 3).

Methods for Identifying periodic genes in P_{GALL}-*CDC20* cells

In previous studies, we have used Pearson correlation as a basis to identify wild-type cell-cycle regulated genes that maintain periodicity in cells depleted for CDK activity (*clb1,2,3,4,5,6* and *cdc28-4*) [1, 9]. While Pearson correlation has successfully identified genes that look the same across two experimental conditions, this method does not directly address what genes in these conditions are periodic. Additionally, Pearson correlation methods score transcript dynamics as similar even if only one cycle of periodic expression agrees and the remaining dynamics do not match. Utilizing this approach may lead to incorrect classification of genes that remain periodic in CDK “on” (Cdc20-depleted) cells.

To identify oscillating gene expression dynamics in non-wild-type cells, each non-wild-type replicate was run through the Lomb-Scargle algorithm at the same period range as wild-type cells [4, 7, 8]. We did not use the deLichtenberg algorithm to analyze these data sets due to the permutation-based method to score periodicity and amplitude [6]. This method makes comparing outputs difficult to do since permuting the data will be different with every experiment.

Cells arrested with persistent mitotic CDK activity do not display any observable periodic oscillations at the microscopic level (P_{GALL}-*CDC20*; Figure S1). Thus, identifying the period of expected oscillations had to be done using transcript dynamics data. To generate a periodic gene list for these datasets, a ten-minute period range centered on 148 minutes was selected because the Lomb-Scargle algorithm classifies the largest number of genes at this period and p-value cutoff. To be consistent with the period range chosen for the wild-type gene lists, a ten-minute period range with a p-value cutoff of $p \leq 0.5$ was selected to generate a periodic gene list.

This method resulted in 420 genes called periodic in cells with stable mitotic CDK activity. Of the periodic genes called from Lomb-Scargle, 208 of those genes are also found in the restrictive periodic list (Additional File 3). The remaining wild-type periodic genes not found to be periodic in CDK "on" cells lose periodicity after the first wave of expression (Figure 2). We were curious about the 214 periodic genes identified in cells with constitutive mitotic CDK activity but not found in the wild-type restrictive periodic gene list. Of these 214 genes, 70 genes were identified by the deLichtenberg algorithm and 14 genes were identified by Lomb-Scargle in wild-type cells. The remaining 130 periodic genes were not identified as periodic in wild-type cells.

Clustering gene lists based on transcript dynamics

Both the DNA replication and spindle assembly checkpoints also arrest with persistent mitotic CDK activity and no observable cell-cycle oscillations ($cdc8^{ts}$ and $P_{GAL1\text{-}10}$-*cse4-353*; Figure S4). The bulk of wild-type periodic genes arrest their oscillatory behavior during these two checkpoints (Figure 4).

To differentiate between the different gene expression behaviors during the checkpoint, the high-confidence wild-type periodic gene list was clustered using affinity propagation [10] with the gene expression dynamics for the DNA replication checkpoint (*cdc8*; Figure S5) or for the spindle assembly checkpoint ($P_{GAL1\text{-}10}$-*cse4-353*; Figure S6). To identify potential transcriptional regulators of each cluster of genes, we performed a transcription factor over-representation analysis based on the *q*-value method [2] using the documented binding information curated by Yeastract [3]. A transcription factor was called over-represented in a cluster if its *q*-value was less than or equal to 0.01. The top five transcription factors are listed next to each cluster (Figures S5 and S6). Additional over-represented transcription factors are listed in

Supplementary Tables 2 and 3. Similar analyses were performed on Cdc20-depleted cells (Figures S3). Additional over-represented transcription factors are listed in Supplementary Table 1.

Identifying periodic behaviors in checkpoint-arrested cells

By visual inspection, we identified two clusters in the DNA replication checkpoint (clusters 7 and 18, Figure S5) and 2 clusters in the spindle assembly checkpoint (clusters 6 and 14, Figure S6) that may exhibit periodic behaviors. Upon visual inspection of the individual genes included in these clusters, only approximately 34 genes remain periodic during the DNA replication checkpoint and 18 genes remain periodic during the spindle assembly checkpoint.

Identifying periodic genes in *$cdc8^{ts}$,P_{GALL}-CDC20,rad53-1* cells

Cells arrested by the *$cdc8^{ts}$* allele, and depleted for CDC20, but without Rad53 signaling display a large number of periodic oscillations. To generate a periodic gene list for these datasets, we used our truncated Rad53 gene lists to 13 time points so that we could compare the p-values generated by periodic algorithms to other datasets (see above). Lomb-Scargle classifies 2399 genes over a periodic range of 92-153 minutes with a p-value of ≤ 0.5. By taking this wide range of periodic behaviors, genes that are not part of the cell-cycle transcription program may be included. To further restrict the gene list, we compared this list to our wild-type restrictive periodic gene list. This method resulted in 343 of the *$cdc8^{ts}$,P_{GALL}-CDC20,rad53-1* periodic genes that were also found in the wild-type periodic gene list. These genes were called the *$cdc8^{ts}$, P_{GALL}-CDC20, rad53-1* cell-cycle periodic gene list (Additional File 3).

Protein Isolation and Immunoblotting

For the immunoblots shown in Figure S1, cells were removed from a synchronous culture at the indicated times post-release and protein was isolated. Briefly, cells were lysed in a modified RIPA lysis buffer (50 mM Tris-HCl, pH 7.5; 250 mM NaCl; 20 mL $Na_2P_4O_7$; 50 mM NaF, 2 mM EDTA, 1% NP-40, 1 mM Na_3VO_4, 1 mM DTT, 0.1 mg/ml PMSF, 1 µg/ml each leupeptin, aprotinin, and pepstatin A) by vortexing with glass beads. Lysates were clarified by high speed centrifugation and protein concentration was measured by A_{280} on a Biophotometer (Eppendorf NA, Hauppauge, NY).

Next, lysates were subjected to SDS-PAGE and transferred to Immobilion-FL PVDF membranes (EMD-Millipore USA, Billerica, MA). Infrared labeled immunoblotting was performed using the following antibodies: mouse anti-HA (Roche Diagnostics, Indianapolis, IN), mouse anti-PSTAIRE (Abcam, Inc., Cambridge, MA), and and IRDye 800 conjugated goat anti-mouse (Li-Cor Biosciences, Lincoln, NE). Labeled PVDF membranes were analyzed with a Li-Cor Odyssey Infrared Imaging System (Li-Cor Biosciences, Lincoln, NE). Signal was quantified using Image J software (National Institutes of Health, USA) and normalized to anti-PSTAIRE.

Supplementary Figures

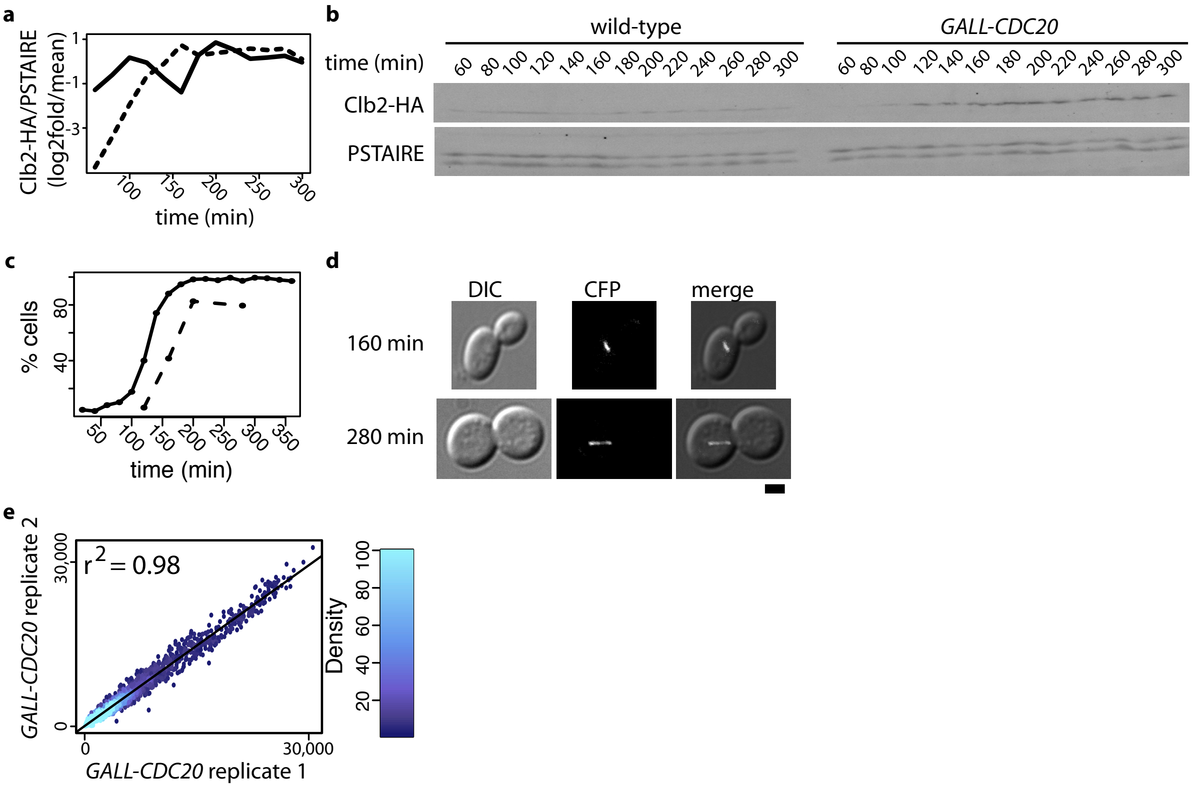

Figure S1 |Additional controls and analyses related to *P*$_{GALL}$*-CDC20* experiments. Dynamics of Clb2 protein levels were measured in P$_{GALL}$*-CDC20;cdc20* and wild-type cells by tagging Clb2 with an HA tag. Quantified levels of Clb2 protein normalized to PSTAIRE and $\log_2$-transformed relative to the mean (**a**). Solid line, wild-type cells; dashed line, P$_{GALL}$-*CDC20;cdc20* cells. Representative blot of three replicate experiments of Clb2-HA levels in wild-type or *P*$_{GALL}$*-CDC20;cdc20* (CDK "on") cells (**b**). Dynamics of budding (solid line) and short spindle formation (dashed line) in G1-synchronized *P*$_{GALL}$*-CDC20* (CDK "on") cells released into dextrose-containing medium (**c**). These cells terminally arrest with a large bud (left, right panel) and short spindle (CFP-tubulin) (middle, right panel) Bar is 5µm (**d**). Reproducibility of *P*$_{GALL}$*-CDC20;cdc20* replicates (**e**). For each probe, the absolute mean expression value/1000 was calculated and plotted (arbitrary units) for each replicate. Coloring of each dot indicates the density of points surrounding the probe in a square with length 500 centered on that point. These data were fitted to a linear model and the corresponding r^2 value is given.

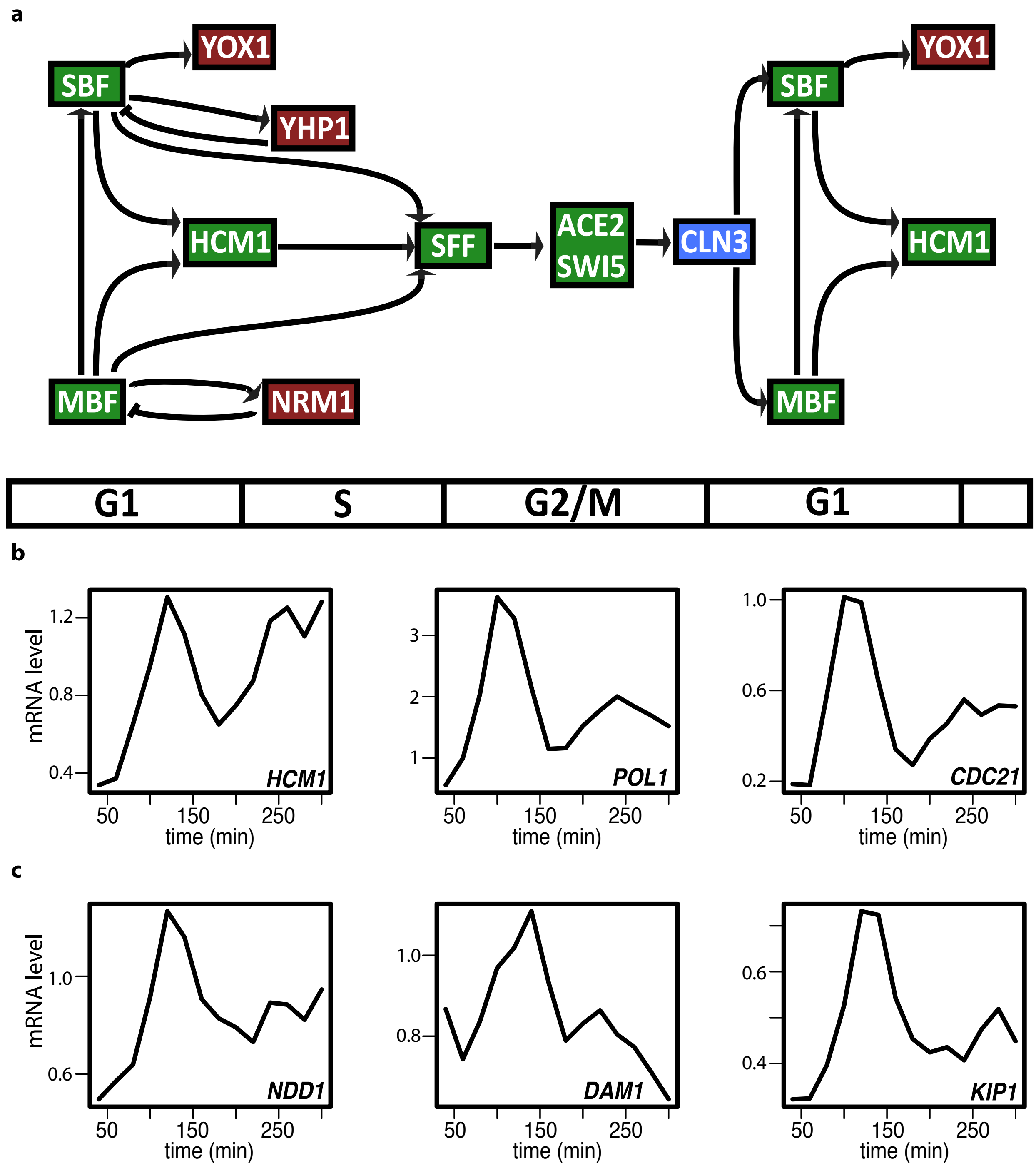

Figure S2 | Additional analyses related to the network transcription factors. Transcription factor network with nodes representing transcription factors; activators are green and repressors are red. Edges indicate that the upstream transcription factor can bind to the promoter of the downstream transcription factor [1] (**a**). Absolute mRNA levels (arbitrary expression units) for canonical MBF targets that continue to oscillate in P_{GALL}-*CDC20;cdc20* (CDK "on") (**b**). Absolute mRNA levels (arbitrary expression units) for canonical Hcm1 targets that continue to oscillate in P_{GALL}-*CDC20;cdc20* (CDK "on") (**c**).

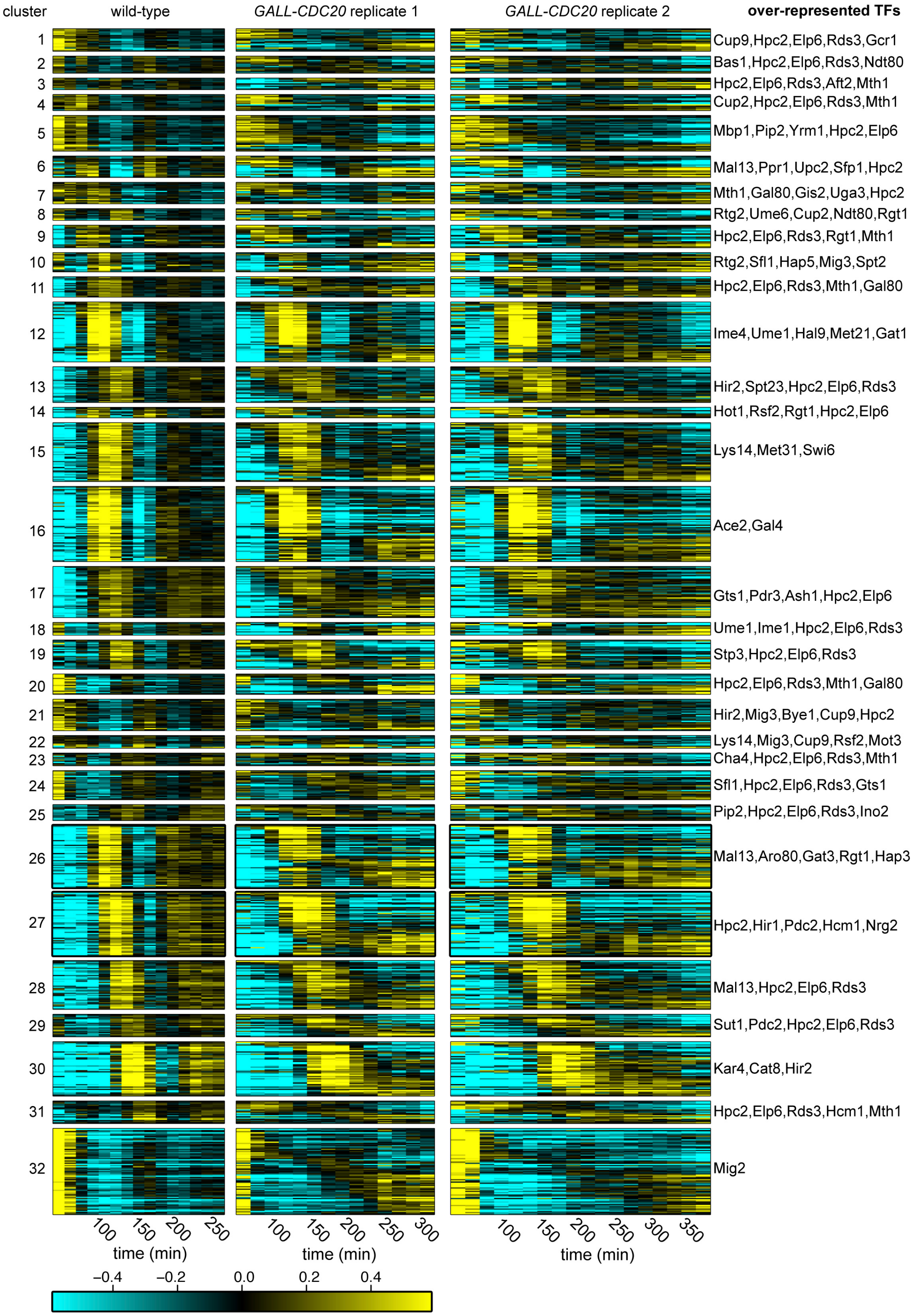
cluster
wild-type
GALL-CDC20 replicate 1
GALL-CDC20 replicate 2
over-represented TFs
1 Cup9,Hpc2,Elp6,Rds3,Gcr1
2 Bas1,Hpc2,Elp6,Rds3,Ndt80
3 Hpc2,Elp6,Rds3,Aft2,Mth1
4 Cup2,Hpc2,Elp6,Rds3,Mth1
5 Mbp1,Pip2,Yrm1,Hpc2,Elp6
6 Mal13,Ppr1,Upc2,Sfp1,Hpc2
7 Mth1,Gal80,Gis2,Uga3,Hpc2
8 Rtg2,Ume6,Cup2,Ndt80,Rgt1
9 Hpc2,Elp6,Rds3,Rgt1,Mth1
10 Rtg2,Sfl1,Hap5,Mig3,Spt2
11 Hpc2,Elp6,Rds3,Mth1,Gal80
12 Ime4,Ume1,Hal9,Met21,Gat1
13 Hir2,Spt23,Hpc2,Elp6,Rds3
14 Hot1,Rsf2,Rgt1,Hpc2,Elp6
15 Lys14,Met31,Swi6
16 Ace2,Gal4
17 Gts1,Pdr3,Ash1,Hpc2,Elp6
18 Ume1,Ime1,Hpc2,Elp6,Rds3
19 Stp3,Hpc2,Elp6,Rds3
20 Hpc2,Elp6,Rds3,Mth1,Gal80
21 Hir2,Mig3,Bye1,Cup9,Hpc2
22 Lys14,Mig3,Cup9,Rsf2,Mot3
23 Cha4,Hpc2,Elp6,Rds3,Mth1
24 Sfl1,Hpc2,Elp6,Rds3,Gts1
25 Pip2,Hpc2,Elp6,Rds3,Ino2
26 Mal13,Aro80,Gat3,Rgt1,Hap3
27 Hpc2,Hir1,Pdc2,Hcm1,Nrg2
28 Mal13,Hpc2,Elp6,Rds3
29 Sut1,Pdc2,Hpc2,Elp6,Rds3
30 Kar4,Cat8,Hir2
31 Hpc2,Elp6,Rds3,Hcm1,Mth1
32 Mig2
100 150 200 250
time (min)
100 150 200 250 300
time (min)
100 150 200 250 300 350
time (min)
−0.4 −0.2 0.0 0.2 0.4

Figure S3 | Additional analyses related to P_{GALL}-*CDC20* time course experiment. Distinguishing different gene expression dynamics in cells depleted of Cdc20. Wild-type periodic genes were clustered by affinity propagation using the first CDK "on" replicate (P_{GALL}-*CDC20*) expression dynamics. Heat maps showing the mRNA levels of clusters in wild-type (left) and in Cdc20-depleted cells (middle; right). Ordering is the same across conditions and replicates. Transcript levels are depicted as $\log_2$-fold change relative to the mean expression. Up to five over-represented transcription factors for each cluster are shown (complete list in Table S1).

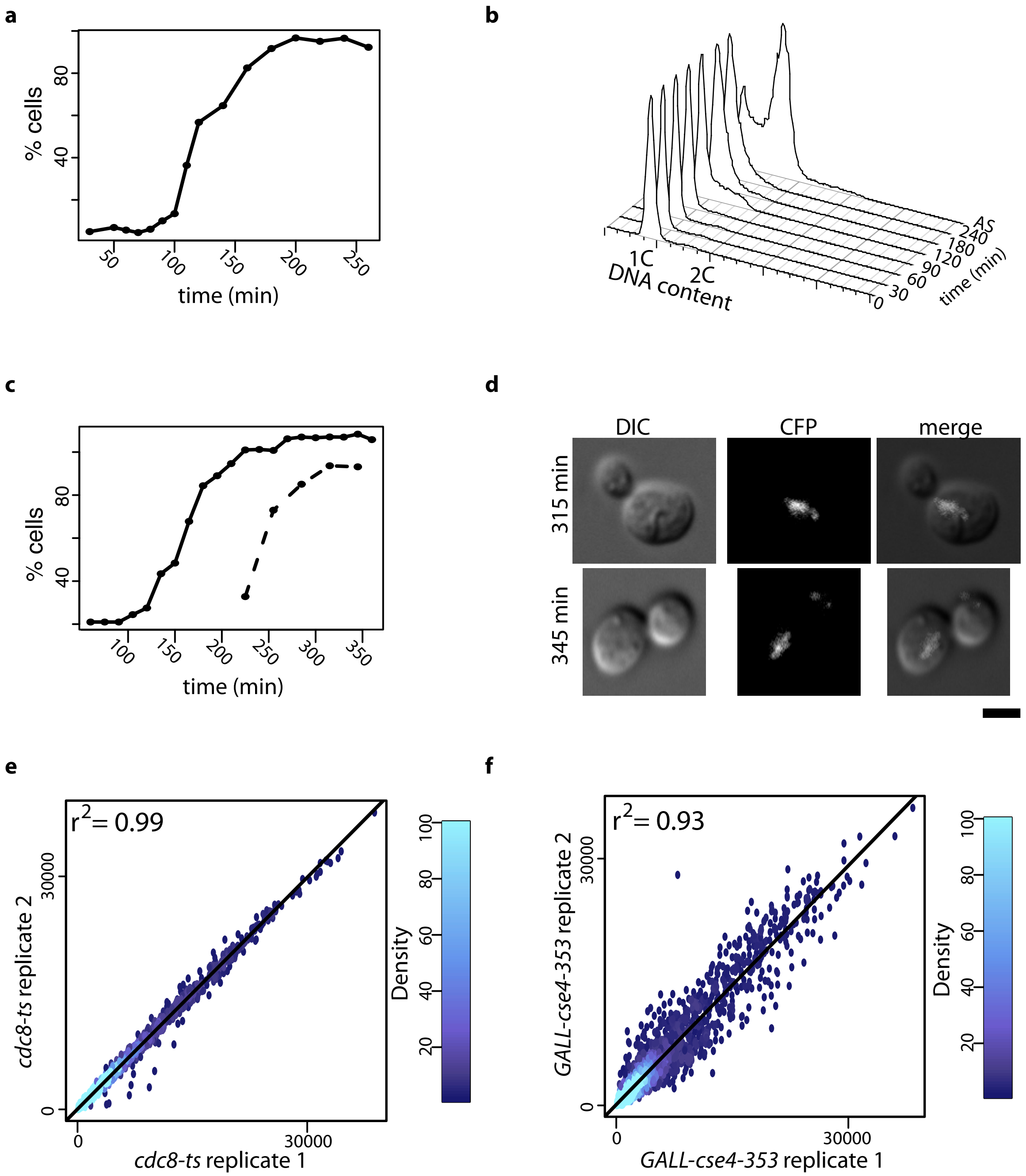

Figure S4 | Additional controls and analyses related to *cdc8-ts* and *P_{GAL1}-cse4-353* experiments. Dynamics of budding in G1-synchronized *$cdc8^{ts}$* cells released into the restrictive temperature (30°C) (**a**). DNA content of *$cdc8^{ts}$* cells during the time series (**b**). Dynamics of budding (solid) and short spindle formation (dashed) of *GAL-cse4-353* cells released into galactose medium to trigger the spindle assembly checkpoint (**c**). These cells terminally arrest with a large bud (left, right panel) and short spindle (CFP-tubulin) (middle, right panel) (**d**). The reproducibility of *$cdc8^{ts}$* (**e**) and *GAL-cse4-353* (**f**) replicate experiments. For each probe, the absolute mean expression value/1000 was calculated and plotted (arbitrary units) for each replicate. Coloring of each dot indicates the density of points surrounding the probe in a square with length 500 centered on that point. These data were fitted to a linear model (black line) and the corresponding r^2 value is given.

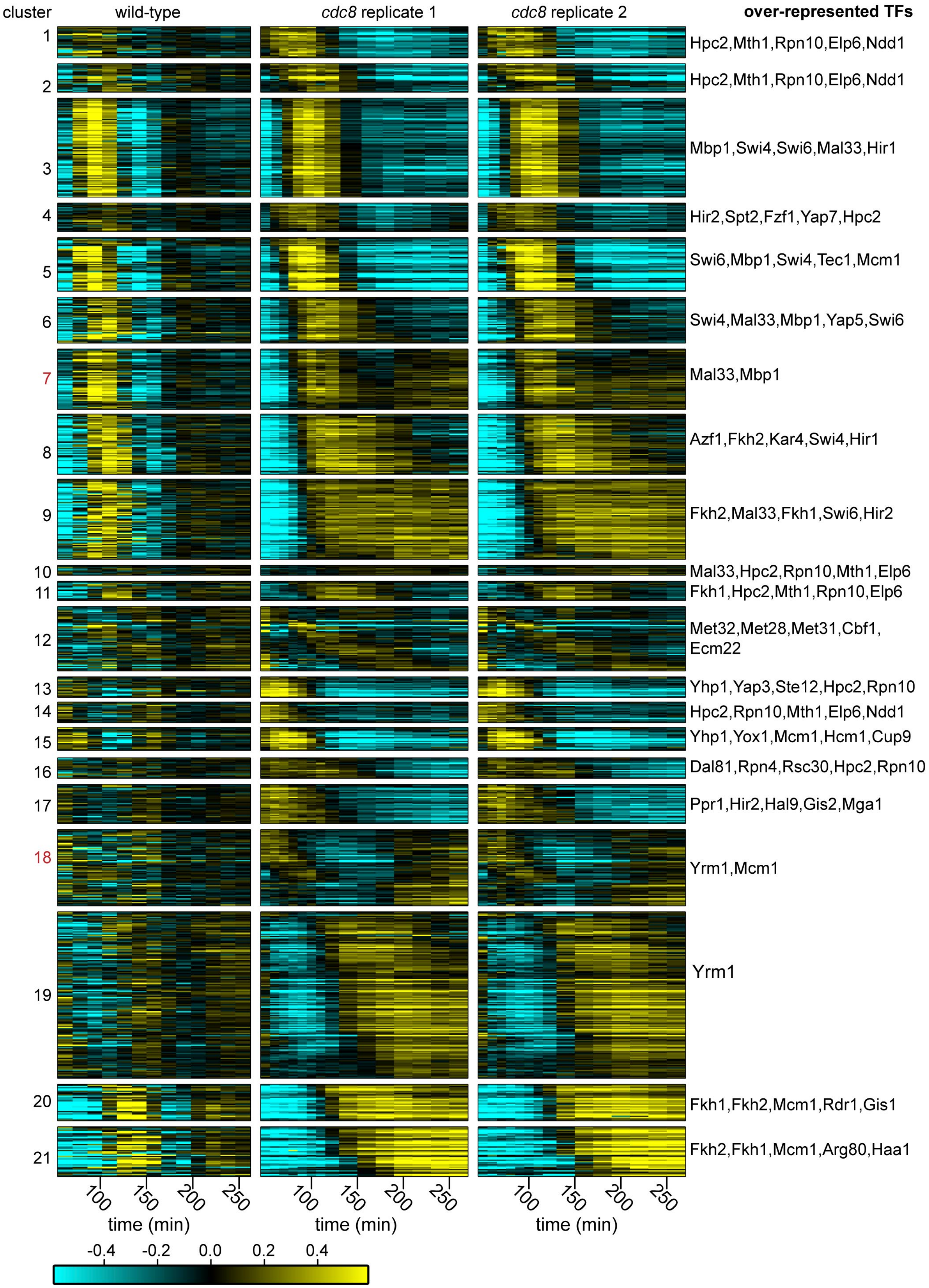
cluster
wild-type
cdc8 replicate 1
cdc8 replicate 2
over-represented TFs
1
Hpc2,Mth1,Rpn10,Elp6,Ndd1
2
Hpc2,Mth1,Rpn10,Elp6,Ndd1
3
Mbp1,Swi4,Swi6,Mal33,Hir1
4
Hir2,Spt2,Fzf1,Yap7,Hpc2
5
Swi6,Mbp1,Swi4,Tec1,Mcm1
6
Swi4,Mal33,Mbp1,Yap5,Swi6
7
Mal33,Mbp1
8
Azf1,Fkh2,Kar4,Swi4,Hir1
9
Fkh2,Mal33,Fkh1,Swi6,Hir2
10
Mal33,Hpc2,Rpn10,Mth1,Elp6
11
Fkh1,Hpc2,Mth1,Rpn10,Elp6
12
Met32,Met28,Met31,Cbf1,
Ecm22
13
Yhp1,Yap3,Ste12,Hpc2,Rpn10
14
Hpc2,Rpn10,Mth1,Elp6,Ndd1
15
Yhp1,Yox1,Mcm1,Hcm1,Cup9
16
Dal81,Rpn4,Rsc30,Hpc2,Rpn10
17
Ppr1,Hir2,Hal9,Gis2,Mga1
18
Yrm1,Mcm1
19
Yrm1
20
Fkh1,Fkh2,Mcm1,Rdr1,Gis1
21
Fkh2,Fkh1,Mcm1,Arg80,Haa1
100
150
200
250
time (min)
-0.4
-0.2
0.0
0.2
0.4

Figure S5 | Additional analyses related to the *cdc8-ts* microarray experiment. Distinguishing different gene expression dynamics during the DNA replication checkpoint. Wild-type periodic genes were clustered by affinity propagation using the first DNA replication checkpoint replicate (*cdc8*^{ts}) expression dynamics. Heat maps showing the mRNA levels of clusters in wild-type (left) and in DNA replication checkpoint-arrested cells (middle, right). Ordering is the same across conditions and replicates. Transcript levels are depicted as $\log_2$-fold change relative to the mean expression. Up to five over-represented transcription factors for each cluster are shown (complete list in Supplementary Table 2).

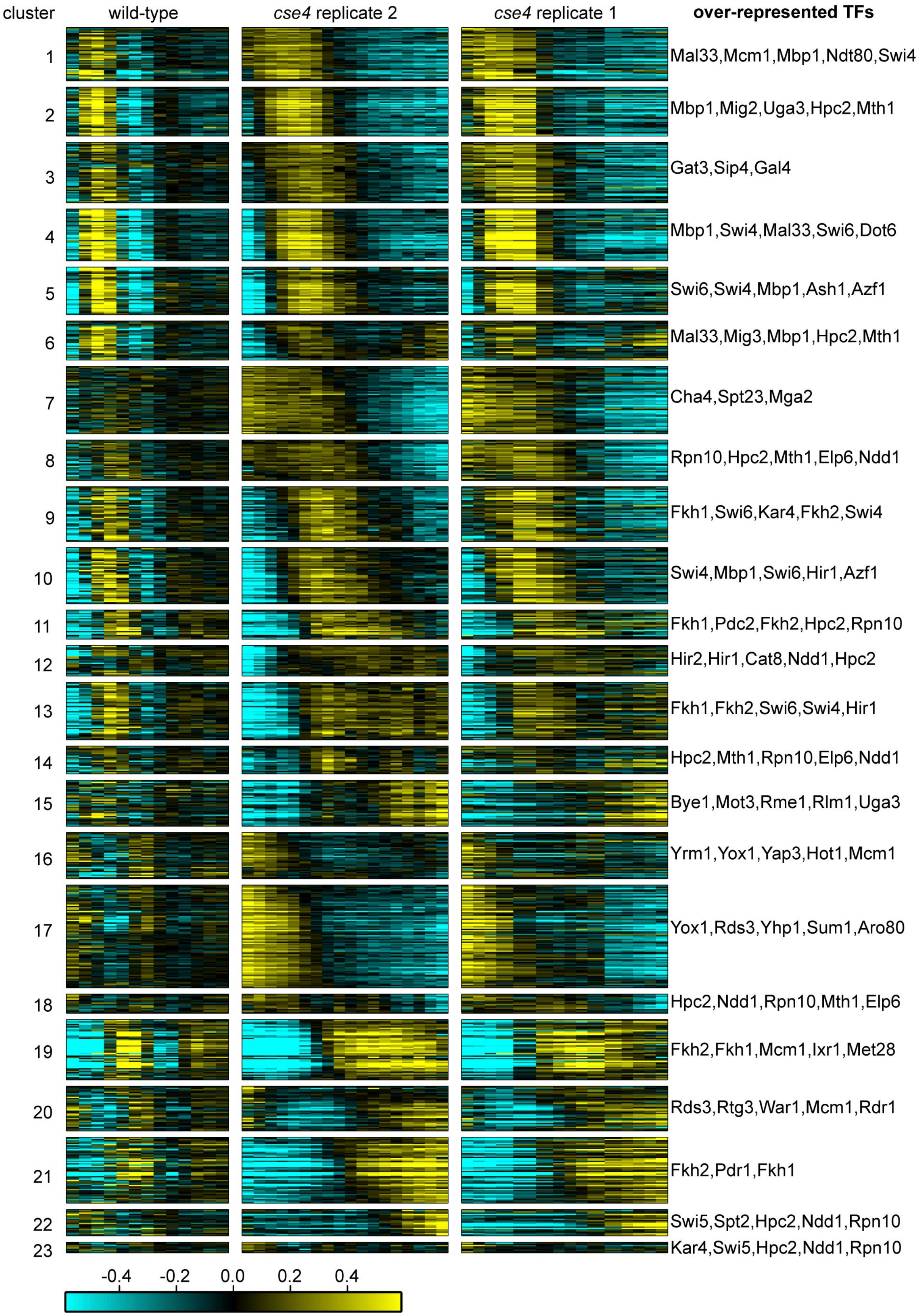
cluster
wild-type
cse4 replicate 2
cse4 replicate 1
over-represented TFs
1 Mal33,Mcm1,Mbp1,Ndt80,Swi4
2 Mbp1,Mig2,Uga3,Hpc2,Mth1
3 Gat3,Sip4,Gal4
4 Mbp1,Swi4,Mal33,Swi6,Dot6
5 Swi6,Swi4,Mbp1,Ash1,Azf1
6 Mal33,Mig3,Mbp1,Hpc2,Mth1
7 Cha4,Spt23,Mga2
8 Rpn10,Hpc2,Mth1,Elp6,Ndd1
9 Fkh1,Swi6,Kar4,Fkh2,Swi4
10 Swi4,Mbp1,Swi6,Hir1,Azf1
11 Fkh1,Pdc2,Fkh2,Hpc2,Rpn10
12 Hir2,Hir1,Cat8,Ndd1,Hpc2
13 Fkh1,Fkh2,Swi6,Swi4,Hir1
14 Hpc2,Mth1,Rpn10,Elp6,Ndd1
15 Bye1,Mot3,Rme1,Rlm1,Uga3
16 Yrm1,Yox1,Yap3,Hot1,Mcm1
17 Yox1,Rds3,Yhp1,Sum1,Aro80
18 Hpc2,Ndd1,Rpn10,Mth1,Elp6
19 Fkh2,Fkh1,Mcm1,Ixr1,Met28
20 Rds3,Rtg3,War1,Mcm1,Rdr1
21 Fkh2,Pdr1,Fkh1
22 Swi5,Spt2,Hpc2,Ndd1,Rpn10
23 Kar4,Swi5,Hpc2,Ndd1,Rpn10
-0.4 -0.2 0.0 0.2 0.4

Figure S6 | Additional analyses related to the *P_{GAL1}-cse4-353* microarray experiment. Distinguishing different gene expression dynamics during the spindle assembly checkpoint. Wild-type periodic genes were clustered by affinity propagation using the second spindle assembly checkpoint replicate (*$P_{GAL1\text{-}10}$-cse4-353*) expression dynamics. Heat maps showing the mRNA levels of clusters in wild-type (left) and in spindle assembly checkpoint-arrested cells (middle, right). Ordering is the same across conditions and replicates. Transcript levels are depicted as $\log_2$-fold change relative to the mean expression. Up to five over-represented transcription factors for each cluster are shown (complete list in Supplementary Table 3).

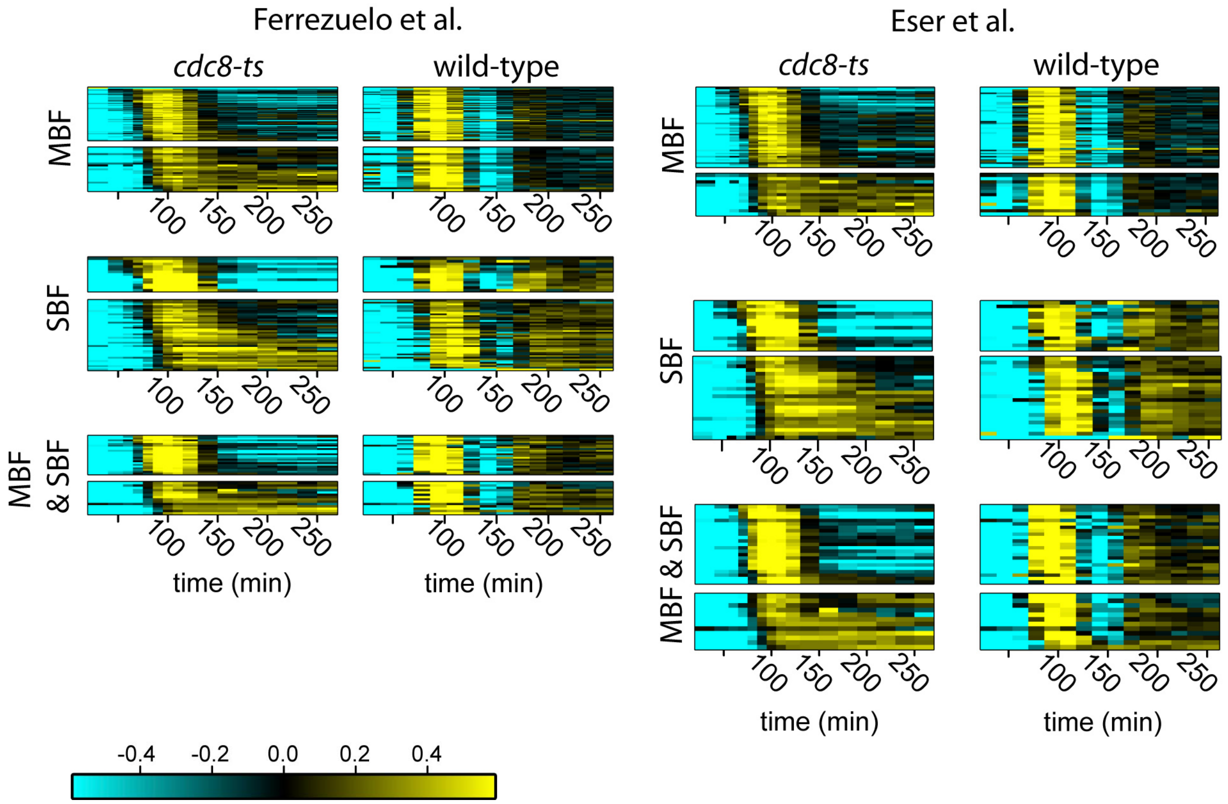

Figure S7 | Additional analysis of transcript levels of SBF- and MBF-regulated genes during the DNA rpelication checkpoint. Heat maps showing mRNA levels of genes shown to be regulated by SBF and/or MBF as determined in previous reports [11, 12] in cells arrested by *cdc8^{ts}* cells (DNA replication checkpoint) and wild-type cells. The same order of genes is shown in all conditions. Transcript levels are depicted as $\log_2$-fold change relative to the mean expression. The identities of genes in each cluster are listed in Additional file 3.

SBF cluster

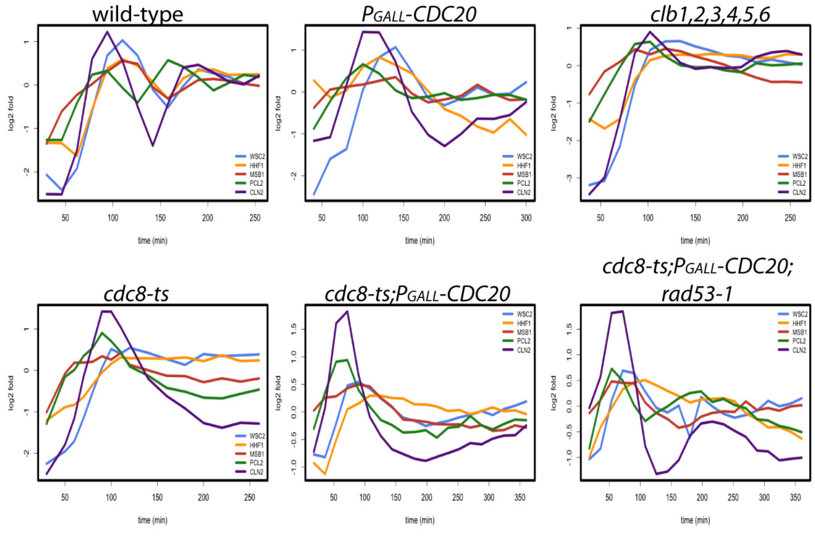

MBF cluster

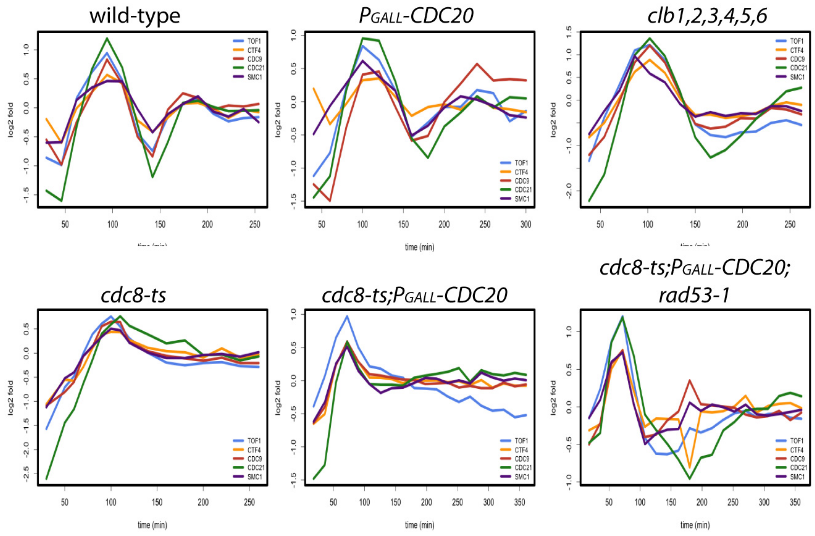

SBF & MBF cluster

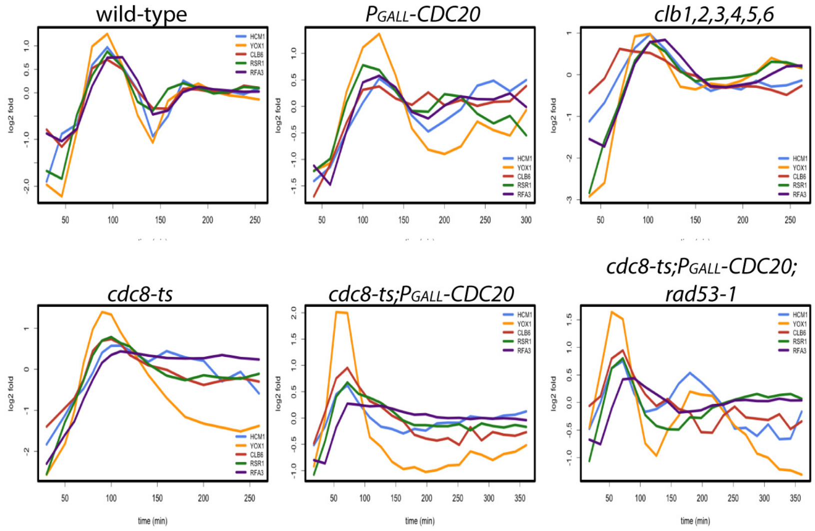

Figure S8 | Additional analysis of transcript levels of co-regulated clusters of genes at G1/S. Line plots of gene expression levels genes are plotted over time from each time course experiment described in this manuscript. Genes were previous described as SBF, MBF, or SBF/MBF regulated by [13]. The expression levels of 5 genes from each cluster are shown for wild-type; *P*$_{GALL}$-*CDC20*; *clb1,2,3,4,5,6*; *cdc8-ts*; *cdc8-ts, P*$_{GALL}$-*CDC20*; and *cdc8-ts, P*$_{GALL}$-*CDC20*, *rad53-1*. Transcript levels are depicted as log_2-fold change relative to the mean expression of each gene.

S phase cluster

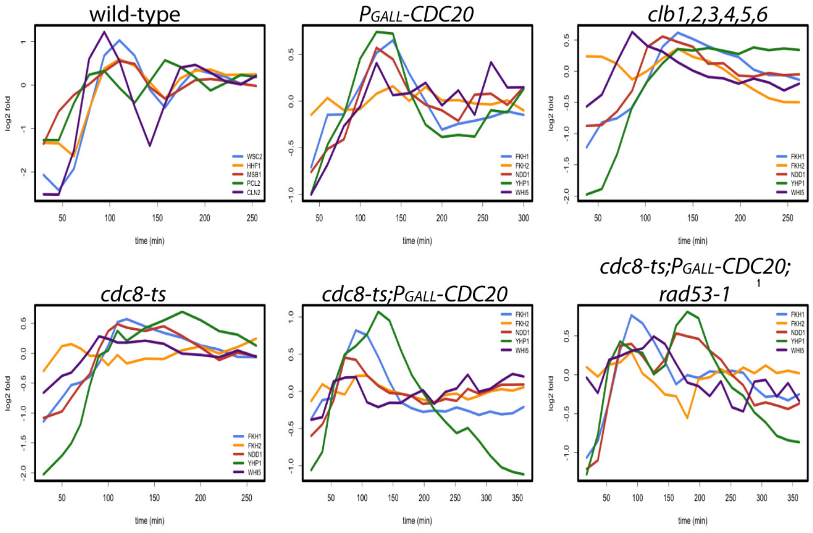

Clb2 cluster

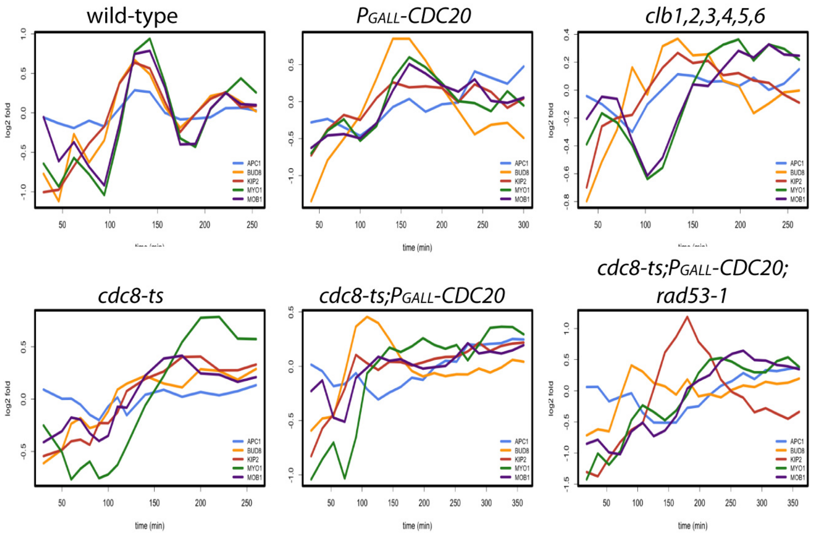

G2/M cluster

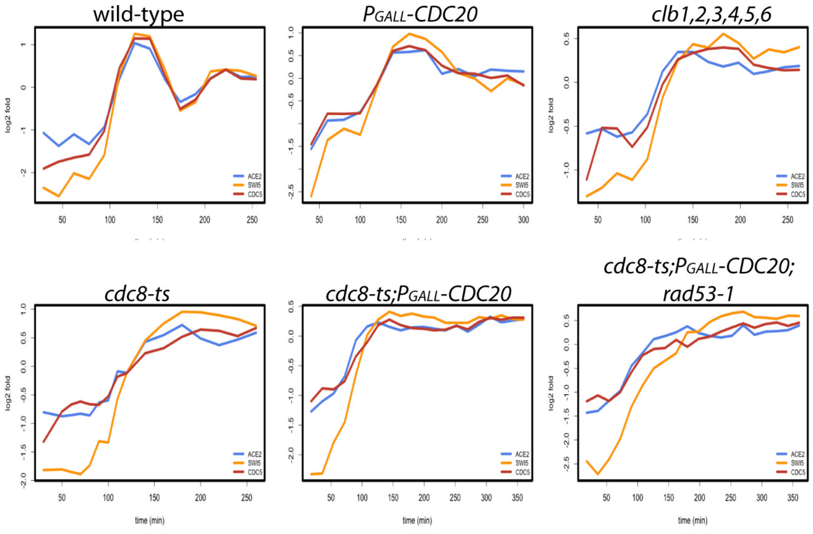

Figure S9 | Additional analysis of transcript levels of co-regulated clusters of genes in S/G2/M. Line plots of gene expression levels genes are plotted over time from each time course experiment described in this manuscript. Genes were previous described as S-phase cluster, Clb2 cluster, or G2/M co-regulated by [13]. The expression levels of 5 genes from the S-phase and Clb2 clusters and 3 genes fro the G2/M cluster are shown for wild-type; P_{GALL}-*CDC20*; *clb1,2,3,4,5,6*; *cdc8-ts*; *cdc8-ts,* P_{GALL}-*CDC20*; and *cdc8-ts,* P_{GALL}-*CDC20*, *rad53-1*. Transcript levels are depicted as $\log_2$-fold change relative to the mean expression of each gene.

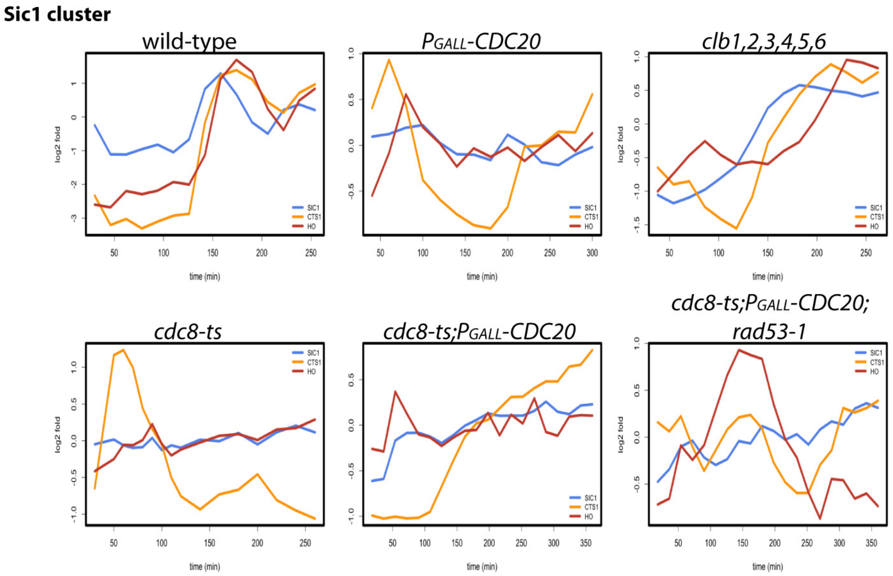

Figure S10 | Additional analysis of transcript levels of co-regulated Sic1 cluster genes. Line plots of gene expression levels genes are plotted over time from each time course experiment described in this manuscript. Genes were previous described as S-phase cluster, Clb2 cluster, or G2/M co-regulated by [13]. The expression levels of 3 genes fro the Sic1 cluster are shown for wild-type; *P_{GALL}-CDC20*; *clb1,2,3,4,5,6*; *cdc8-ts*; *cdc8-ts, P_{GALL}-CDC20*; and *cdc8-ts, P_{GALL}-CDC20*, *rad53-1*. Transcript levels are depicted as $\log_2$-fold change relative to the mean expression of each gene.

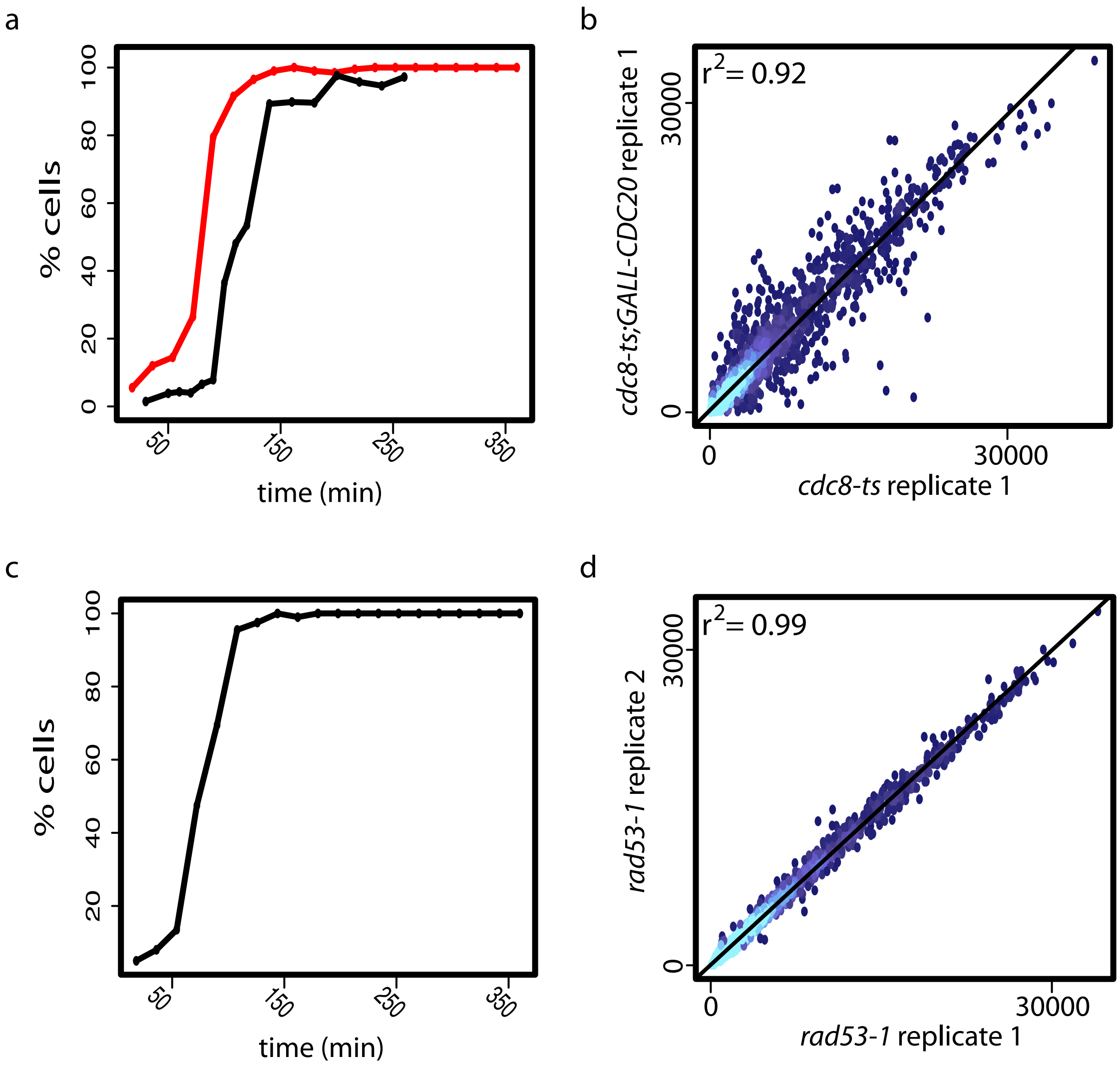

Figure S11 | Additional controls and analyses related to *cdc8-ts cdc20 P_{GALL}-CDC20* and *rad53-1 cdc8-ts cdc20 P_{GALL}-CDC20* experiments (Figure 6). Dynamics of budding in G1-synchronized *$cdc8^{ts}$ cdc20 P_{GALL}-CDC20* cells released into the restrictive temperature (30°C), red (black is *$cdc8^{ts}$*) (**a**). The similarities between *$cdc8^{ts}$ cdc20 P_{GALL}-CDC20* and *$cdc8^{ts}$* (**b**). Dynamics of budding *rad53-1 $cdc8^{ts}$ cdc20 P_{GALL}-CDC20* (**c**). The reproducibility of *rad53-1 $cdc8^{ts}$ cdc20 P_{GALL}-CDC20* replicate experiments (**d**). For each probe, the absolute mean expression value/1000 was calculated and plotted (arbitrary units) for each replicate (**b and d**). Coloring of each dot indicates the density of points surrounding the probe in a square with length 500 centered on that point. These data were fitted to a linear model (black line) and the corresponding r^2 value is given.

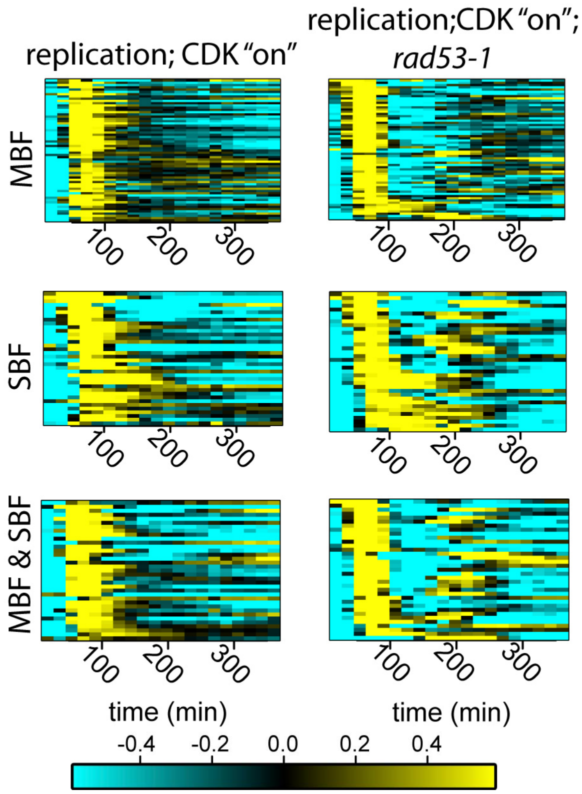

Figure S12 | Additional analysis of transcript dynamics of SBF- and MBF- regulated genes during the DNA replication checkpoint with and without Rad53 activity (Figure 6). Distinguishing different gene expression dynamics of SBF- and MBF regulated genes in DNA replication checkpoint arrested cells with and without Rad53 activity. Gene lists were taken from analyses generated Eser et al [11, 12]. Heat maps showing the mRNA levels of clusters in DNA replication checkpoint with Rad53 activity (left) and without Rad53 activity (right). Ordering is the same across conditions (Additional File 3). Transcript levels are depicted as log_2-fold change relative to the mean expression.

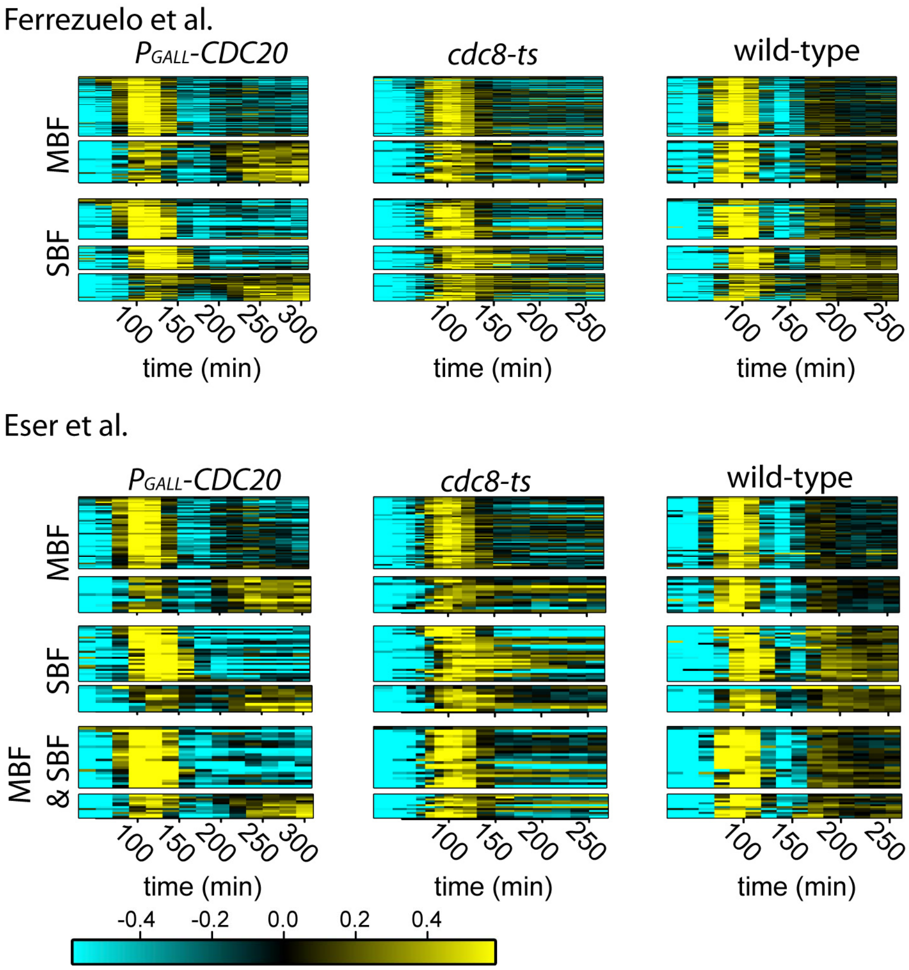

Figure S13 | Additional analysis of transcript dynamics of SBF- and MBF- regulated genes during the DNA replication checkpoint and in the absence of Cdc20 activity. Distinguishing different gene expression dynamics of SBF- and MBF regulated genes in DNA replication and CDK "on" arrested cells. Gene lists were taken from analyses generated in one of two previous studies [11, 12]. Heat maps showing the mRNA levels of clusters in CDK "on" (P_{GALL}-*CDC20*) (left), DNA replication checkpoint *(cdc8-ts*) (middle), and normally-cycling cells (right). Ordering is the same across conditions, and genes within each cluster are as found in figure 3d,e and are listed in Additional file 3. Transcript levels are depicted as $\log_2$-fold change relative to the mean expression.

Table S1 | Over-represented transcription factors in P_{GALL}-*CDC20* (CDK "on") clusters. Each row corresponds to a CDK "on" cluster (1-21). Each cluster has a set of corresponding transcription factors with *q*-values ≤ 0.01, sorted from most to least significant.

Cluster	Over-represented TFs with Q-value < 0.01
1	Cup9,Hpc2,Elp6,Rds3,Gcr1,Mth1,Aca1,Gal80,Pip2
2	Bas1,Hpc2,Elp6,Rds3,Ndt80,Sko1,Mth1,Aca1,Gal80,Hir1,Ume1
3	Hpc2,Elp6,Rds3,Aft2,Mth1,Aca1,Gal80,Thi2,Oaf2,Hir1,Ume1,Lys14,Rtg2,Rdr1
4	Cup2,Hpc2,Elp6,Rds3,Mth1,Aca1,Gal80,Aro80,Hir1,Ume1,Sip4
5	Mbp1,Pip2,Yrm1,Hpc2,Elp6,Rds3,Oaf1
6	Mal13,Ppr1,Upc2,Sfp1,Hpc2,Elp6,Rds3,Arr1,Zap1,YPR015C,Mth1,Aca1,Gal80,Gat1
7	Mth1,Gal80,Gis2,Uga3,Hpc2,Elp6,Rds3,Gat3,Ecm22,Aca1,Rds1
8	Rtg2,Ume6,Cup2,Ndt80,Rgt1,Mig1,Elp6,Rds3,Ixr1,Phd1,Mth1,Gal80,Aca1, Sps18,Oaf1,Crz1, Hir1,Ume1,Yrr1,Lys14,Rdr1,Rtg1
9	Hpc2,Elp6,Rds3,Rgt1,Mth1,Gal80,Aca1,Hir1,Ume1
10	Rtg2,Sfl1,Hap5,Mig3,Spt2,Rsf2,Hpc2,Elp6,Rds3,Mot3,Rgt1,Hap3,Hal9,Mth1,Gal80, Aca1,Hap2,Aft1,Mig2
11	Hpc2,Elp6,Rds3,Mth1,Gal80,Aca1
12	Ime4,Ume1,Hal9,Met31,Gat1
13	Hir2,Spt23,Hpc2,Elp6,rds3,Ppr1
14	Hot1,Rsf2,Rgt1,Hpc2,Elp6,Rds3,Sip4,Mth1,Gal80,Aca1,Ume1,Hir1,Rtg2,Lys14,Rdr1, Sum1, Azf1
15	Lys14,Met31,Swi6
16	Ace2,Gal4
17	Gts1,Pdr3,Ash1,Hpc2,Elp6,Rds3
18	Ume1,Ime1,Hpc2,Elp6,Rds3,Rds1,Mac1,Rgm1,Mth1,Gal80,Aca1,Hac1,Hir1,Lys14,Rtg2,Rdr1
19	Stp3,Hpc2,Elp6,Rds3
20	Hpc2,Elp6,Rds3,Mth1,Gal80,Aca1
21	Hir2,Mig3,Bye1,Cup9,Hpc2,Elp6,Rds3
22	Lys14,Mig3,Cup9,Rsf2,Mot3,Put3,Hpc2,Elp6,Rds3,Rim101,Mig2,Mth1,Gal80,Aca1 ,Phd1,Hac1,Ume1,Hir1,Mga1,Rtg2,Rdr1
23	Cha4,Hpc2,Elp6,Rds3,Mth1,Gal80,Aca1,Aro80,Ume1,Hir1,Lys14,Rtg2,Rdr1
24	Sfl1,Hpc2,Elp6,Rds3,Gts1
25	Pip2,Hpc2,Elp6,Rds3,Ino2,Rds2,Mth1,Gal80,Aca1,Cst6,Sps18,Ume1,Hir1,Sut1,Gcr1
26	Mal13,Aro80,Gat3,Rgt1,Hap3,Rme1
27	Hpc2,Hir1,Pdc2,Hcm1,Nrg2
28	Mal13,Hpc2,Elp6,Rds3
29	Sut1,Pdc2,Hpc2,Elp6,Rds3,Mth1,Gal80,Aca1
30	Kar4,Cat8,Hir2
31	Hpc2,Elp6,Rds3,Hcm1,Mth1,Gal80,Aca1
32	Mig2

Table S2 | Over-represented transcription factors in *cdc8*^{ts} (DNA replication checkpoint) clusters. Each row corresponds to a DNA replication checkpoint cluster (1-21). Each cluster has a set of corresponding transcription factors with *q*-values ≤ 0.01, sorted from most to least significant

Cluster	Over-represented TFs with Q-value < 0.01
1	Hpc2,Mth1,Rpn10,Elp6,Ndd1,Mdl2,Fzf1
2	Hpc2,Mth1,Rpn10,Elp6,Ndd1,Mdl2,Opi1,Swi6,Ndt80
3	Mbp1,Swi4,Swi6,Mal33,Hir1,Ume1
4	Hir2,Spt2,Fzf1,Yap7,Hpc2,Mth1,Rpn10,Elp6,Ndd1,Mdl2,Mga2
5	Swi6,Mbp1,Swi4,Tec1,Mcm1,Smp1,Ndt80,Rlm1,Ash1,Gal4,Ime1,Ste12,Azf1,Xbp1,Hal9
6	Swi4,Mal33,Mbp1,Yap5,Swi6,Pdc2,Yrm1,Hpc2,Mth1,Elp6,Ndd1,Mdl2,Bye1
7	Mal33,Mbp1
8	Azf1,Fkh2,Kar4,Swi4,Hir1,Swi6,Mbp1,Fkh1,Stp3
9	Fkh2,Mal33,Fkh1,Swi6,Hir2,Mbp1,Swi4,Hir1
10	Mal33,Hpc2,Rpn10,Mth1,Elp6,Ndd1,Mdl2,Pdr1,Gal80,Rds3,Hir1,Imp2',Ume1,Aca1,Swi6, Tye7,Lys14,Rtg2,Mal13
11	Fkh1,Hpc2,Mth1,Rpn10,Elp6,Ndd1,Mdl2,Arg81,Dal82,Gal80,Rds3,Cha4
12	Met32,Met28,Met31,Cbf1,Ecm22
13	Yhp1,Yap3,Ste12,Hpc2,Rpn10,Mth1,Elp6,Ndd1,Mdl2,Mga2,Mcm1,Swi5,Gal80,Rds3,Phd1
14	Hpc2,Rpn10,Mth1,Elp6,Ndd1,Mdl2,Ndt80,Gal80,Rds3
15	Yhp1,Yox1,Mcm1,Hcm1,Cup9,Azf1,Kar4,Hpc2,Rpn10,Mth1,Elp6,Ndd1,Mdl2,Flo8,Gal80, Rds3
16	Dal81,Rpn4,Rsc30,Hpc2,Rpn10,Mth1,Elp6,Ndd1,Mdl2,Gal80,Rds3,Hap3,Rph1
17	Ppr1,Hir2,Hal9,Gis2,Mga1,Hpc2,Rpn10,Mth1,Elp6,Ndd1,Mdl2,Dal80
18	Yrm1,Mcm1
19	Yrm1
20	Fkh1,Fkh2,Mcm1,Rdr1,Gis1,Hpc2,Rpn10,Mth1,Elp6,Mdl2,Flo8,Fzf1
21	Fkh2,Fkh2,Mcm1,Arg80,Haa1,Arg81,Hir2,Mbp1,YPR015c,Yhp1,Cha4,Thi2,Ndd1,Hpc2, Rpn10,Mth1,Elp6,Mdl2

Table S3 | Over-represented transcription factors in *P_{GAL}-cse4-353* (spindle assembly checkpoint) clusters. Each row corresponds to a spindle assembly checkpoint cluster (1-21). Each cluster has a set of corresponding transcription factors with *q*-values ≤ 0.01, sorted from most to least significant.

Cluster	Over-represented TFs with Q-value < 0.01
1	Mal33,Mcm1,Mbp1,Ndt80,Swi4,Rds1,Ino4,Swi6,Ime1,Hpc2,Mth1,Rpn10,Elp6,Ndd1,Mdl2
2	Mbp1,Mig2,Uga3,Hpc2,Mth1,Rpn10,Elp6,Ndd1,Mdl2,Mal33
3	Gat3,Sip4,Gal4
4	Mbp1,Swi4,Mal33,Swi6,Dot6,Hpc2,Mth1,Elp6,Ndd1,Mdl2
5	Swi6,Swi4,Mbp1,Ash1,Azf1,Mal33,Ume1,Tec1,Rlm1,Mcm1,Hal9,Rpn10,Hpc2,Mth1,Elp6, Ndd1,Mdl2
6	Mal33,Mig3,Mbp1,Hpc2,Mth1,Rpn10,Elp6,Ndd1,Mdl2
7	Cha4,Spt23,Mga2
8	Rpn10,Hpc2,Mth1,Elp6,Ndd1,Mdl2
9	Fkh1,Swi6,Kar4,Fkh2,Swi4,Rpn10,Hpc2,Mth1,Elp6,Ndd1,Mdl2
10	Swi4,Mbp1,Swi6,Hir1,Azf1,Hir2,Rpn10,Mth1,Elp6,Ndd1,Mdl2
11	Fkh1,Pdc2,Fkh2,Hpc2,Rpn10,Mth1,Elp6,Mdl2,Kar4,Ndt80
12	Hir2,Hir1,Cat8,Ndd1,Hpc2,Rpn10,Mth1,Elp6,Mdl2,Azf1,Swi6
13	Fkh1,Fkh2,Swi6,Swi4,Hir1,Kar4,Ash1,Mbp1,Phd1,Ste12,Stp3,Hcm1,Yrm1
14	Hpc2,Mth1,Rpn10,Elp6,Ndd1,Mdl2,Gal80,Rds3
15	Bye1,Mot3,Rme1,Rlm1,Uga3,Sps18,Cst6,Hpc2,Mth1,Rpn10,Elp6,Ndd1,Mdl2
16	Yrm1,Yox1,Yap3,Hot1,Mcm1,Pdr8,Gis2,Hpc2,Mth1,Rpn10,Elp6,Ndd1,Mdl2
17	Yox1,Rds3,Yhp1,Sum1,Aro80
18	Hpc2,Ndd1,Rpn10,Mth1,Elp6,Mdl2,Rds3,Gal80,Cha4,Aro80,Hir1,Imp2'
19	Fkh2,Fkh1,Mcm1,Ixr1,Met28,Arg80,Rdr1,Ino2, Haa1,Met32,Azf1,Cha4
20	Rds3,YPR015C,Rtg3,War1,Mcm1,Rdr1,Cst6,Haa1,Ash1,Pdr1,Flo8,Crz1,Rlm1,Bye1,Cup9, Hpc2,Mth1,Rpn10,Elp6,Ndd1,Mdl12,Gzf3
21	Fkh2,Pdr1,Fkh1
22	Swi5,Spt2,Hpc2,Ndd1,Rpn10,Mth1,Elp6,Mdl2,Rds3,Gal80,Ace2
23	Kar4,Swi5,Hpc2,Ndd1,Rpn10,Mth1,Elp6,Mdl2,Thi2,Rgm1,Rds3,Gal80,Hir1,Imp2',Ume1, Aca1,Azf1,Ifh1,Lys14,Rtg2,Mal13

Table S4 | Strain list. Relevant strains used in this study to generate data.

strain	relavent genotype	reference
15Da-	*MATa; bar1;ade1;his2;leu2-3112;trp1-1;ura3Δ3*	
SBY1621	*MATa; bar1;cdc20::LEU2;Pgall-CDC20::ADE2;CFP-TUB1::URA3*	DiTalia, et al.[14]
SBY353	*MATa; BAR1;cdc8*^{ts}	Hartwell [15]
SBY1592	*MATa;Pgal-cse4-353::TRP1;CFP-TUB1::URA3*	Collins, et al.[16]
SBY2124	*MATa;bar1;cdc20::LEU2;Pgall-CDC20::ADE2;CFP-TUB1::URA3;CLB2-HA:kanMX6*	This study
SBY1258	*MATa;bar1;CLB2-HA:kanMX6*	This study
SBY2201	*MATa; bar1;cdc8*^{ts}*;cdc20::LEU2;Pgall-CDC20::ADE2;CFP-TUB1::URA3*	This study
SBY2229	*MATa; bar1;rad53-1;cdc8*^{ts}*;cdc20::LEU2;Pgall-CDC20::ADE2;CFP-TUB1::URA3*	This study

References

1. Orlando DA, Lin CY, Bernard A, Wang JY, Socolar JE, Iversen ES, Hartemink AJ, Haase SB: **Global control of cell-cycle transcription by coupled CDK and network oscillators.** *Nature* 2008, **453:**944-947.
2. Storey JD: **A direct approach to false discovery rates.** *Journal of the Royal Statistical Society Series B-Statistical Methodology* 2002, **64:**479-498.
3. Teixeira MC, Monteiro P, Jain P, Tenreiro S, Fernandes AR, Mira NP, Alenquer M, Freitas AT, Oliveira AL, Sa-Correia I: **The YEASTRACT database: a tool for the analysis of transcription regulatory associations in Saccharomyces cerevisiae.** *Nucleic Acids Res* 2006, **34:**D446-451.
4. Lomb NR: **Least-Squares Frequency-Analysis of Unequally Spaced Data.** *Astrophysics and Space Science* 1976, **39:**447-462.
5. Orlando DA, Lin CY, Bernard A, Iversen ES, Hartemink AJ, Haase SB: **A probabilistic model for cell cycle distributions in synchrony experiments.** *Cell Cycle* 2007, **6:**478-488.
6. de Lichtenberg U, Jensen LJ, Fausboll A, Jensen TS, Bork P, Brunak S: **Comparison of computational methods for the identification of cell cycle-regulated genes.** *Bioinformatics* 2005, **21:**1164-1171.
7. Glynn EF, Chen J, Mushegian AR: **Detecting periodic patterns in unevenly spaced gene expression time series using Lomb-Scargle periodograms.** *Bioinformatics* 2006, **22:**310-316.
8. Scargle JD: **Studies in astronomical time series analysis. II-Statistical aspects of spectral analysis of unevenly spaced data.** *Astrophysical Journal* 1982, **263:**835-853.
9. Simmons Kovacs LA, Mayhew MB, Orlando DA, Jin Y, Li Q, Huang C, Reed SI, Mukherjee S, Haase SB: **Cyclin-dependent kinases are regulators and effectors of oscillations driven by a transcription factor network.** *Mol Cell*, **45:**669-679.
10. Frey BJ, Dueck D: **Clustering by passing messages between data points.** *Science* 2007, **315:**972-976.
11. Eser U, Falleur-Fettig M, Johnson A, Skotheim JM: **Commitment to a cellular transition precedes genome-wide transcriptional change.** *Mol Cell* 2011, **43:**515-527.
12. Ferrezuelo F, Colomina N, Futcher B, Aldea M: **The transcriptional network activated by Cln3 cyclin at the G1-to-S transition of the yeast cell cycle.** *Genome Biol*, **11:**R67.
13. Haase SB, Wittenberg C: **Topology and control of the cell-cycle-regulated transcriptional circuitry.** *Genetics* 2014, **196:**65-90.
14. Di Talia S, Wang H, Skotheim JM, Rosebrock AP, Futcher B, Cross FR: **Daughter-specific transcription factors regulate cell size control in budding yeast.** *PLoS Biol* 2009, **7:**e1000221.
15. Hartwell LH, Culotti J, Pringle JR, Reid BJ: **Genetic control of the cell division cycle in yeast.** *Science* 1974, **183:**46-51.

16. Collins KA, Camahort R, Seidel C, Gerton JL, Biggins S: **The overexpression of a Saccharomyces cerevisiae centromeric histone H3 variant mutant protein leads to a defect in kinetochore biorientation.** *Genetics* 2007, **175:**513-525.
